# Radiomics-based machine learning analysis and characterization of breast lesions with multiparametric diffusion-weighted MR

**DOI:** 10.1186/s12967-021-03117-5

**Published:** 2021-10-24

**Authors:** Kun Sun, Zhicheng Jiao, Hong Zhu, Weimin Chai, Xu Yan, Caixia Fu, Jie-Zhi Cheng, Fuhua Yan, Dinggang Shen

**Affiliations:** 1 Department of Radiology, Ruijin Hospital, Shanghai Jiaotong University School of Medicine, Shanghai, China; 2 Department of Diagnostic Imaging, Alpert Medical School of Brown University, Providence, USA; 3 Scientific Marketing, Siemens Shanghai Magnetic Resonance Ltd., Shanghai, China; 4 MR Application Development, Siemens Shenzhen Magnetic Resonance Ltd., Shenzhen, China; 5 Department of Research and Development, Shanghai United Imaging Intelligence Co., Ltd., Shanghai, China; 6 School of BME, Shanghai Tech University, Shanghai, China

**Keywords:** Breast cancer, Diffusion-weighted MRI, Machine learning, Random forest

## Abstract

**Background:**

This study aimed to evaluate the utility of radiomics-based machine learning analysis with multiparametric DWI and to compare the diagnostic performance of radiomics features and mean diffusion metrics in the characterization of breast lesions.

**Methods:**

This retrospective study included 542 lesions from February 2018 to November 2018. One hundred radiomics features were computed from mono-exponential (ME), biexponential (BE), stretched exponential (SE), and diffusion-kurtosis imaging (DKI). Radiomics-based analysis was performed by comparing four classifiers, including random forest (RF), principal component analysis (PCA), L1 regularization (L1R), and support vector machine (SVM). These four classifiers were trained on a training set with 271 patients via ten-fold cross-validation and tested on an independent testing set with 271 patients. The diagnostic performance of the mean diffusion metrics of ME (mADC_all b_, mADC_0–1000_), BE (mD, mD^*^, mf), SE (mDDC, mα), and DKI (mK, mD) were also calculated for comparison. The area under the receiver operating characteristic curve (AUC) was used to compare the diagnostic performance.

**Results:**

RF attained higher AUCs than L1R, PCA and SVM. The AUCs of radiomics features for the differential diagnosis of breast lesions ranged from 0.80 (BE_D*) to 0.85 (BE_D). The AUCs of the mean diffusion metrics ranged from 0.54 (BE_mf) to 0.79 (ME_mADC_0–1000_). There were significant differences in the AUCs between the mean values of all diffusion metrics and radiomics features of AUCs (all *P* < 0.001) for the differentiation of benign and malignant breast lesions. Of the radiomics features computed, the most important sequence was BE_D (AUC: 0.85), and the most important feature was FO-10 percentile (Feature Importance: 0.04).

**Conclusions:**

The radiomics-based analysis of multiparametric DWI by RF enables better differentiation of benign and malignant breast lesions than the mean diffusion metrics.

**Supplementary Information:**

The online version contains supplementary material available at 10.1186/s12967-021-03117-5.

## Background

Breast MRI is widely used for breast cancer diagnosis and treatment evaluation [1]. Dynamic contrast-enhanced (DCE) sequences with the use of a contrast agent can provide both morphological and hemodynamic cues for lesion diagnosis. However, a higher false-positive rate and background parenchymal enhancement limit the diagnostic specificity of DCE [2, 3].

Diffusion-weighted imaging (DWI), a noninvasive contrast agent-free method, has been established for breast MR imaging and could improve the diagnostic specificity of lesions suspicious for breast cancer [4, 5]. Conventional DWI (mono-exponential model) with 2 to 3 b-values for the measurement of the apparent diffusion coefficient (ADC) is the most commonly used diffusion fitting model for the characterization of breast lesions [6]. Furthermore, several studies have suggested that biexponential (BE), stretched-exponential (SE), or diffusion kurtosis imaging (DKI), fitting with multi-b-value sequences, could provide more accurate information about water diffusion [7–10]. Le Bihan et al. [11, 12] proposed the intravoxel incoherent motion (IVIM) model, a kind of BE fitting, to separately calculate fast and slow diffusion components. The SE model was introduced by Bennett et al. [13] to depict the heterogeneity of intravoxel diffusion rates and the distributed diffusion effect. The DKI model was proposed by Jensen et al. [14] to reflect the complexity of the microenvironment. Since DWI with different fitting models may demonstrate different aspects of tissue properties [7, 15, 16], informative radiomics features could be derived from these models to better characterize breast lesions.

Radiomics-based analysis profiles lesions with extensive morphological and textural features for latter classification models to attain better differential diagnosis, prognosis prediction, and tumor subtype diagnosis, etc. [17–20]. Previous radiomics studies [21–23] of breast lesions focused more on the modalities of T2WI and DCE. Fewer studies [17, 24, 25] have considered the value of multiparametric DWI. However, to our knowledge, no study comparing the radiomics features of these different diffusion imaging approaches in the differentiation of breast lesions has been conducted. Further study of multiparametric DWI using more effective machine learning methods is needed to better understand their predictive value in breast cancer diagnosis.

We hypothesized that the diagnostic accuracy of breast lesions using multi-b-value sequences combined with ME, BE, SE and DKI can be improved by radiomics-based analysis. The purpose of this study is to compare radiomics features and the mean values of diffusion metrics in the assessment of breast lesions with four machine learning methods, i.e., random forest (RF), L1 regularization combined with linear regression (L1R-LR), principal component analysis combined with linear regression (PCA-LR), and support vector machine (SVM).

## Methods

### Study design and patient selection

This retrospective study was performed with a prospectively acquired data set with institutional and governmental review board approval. The local Institutional Review Board (IRB) approved this study. Written informed consent was obtained from each participant. From February 2018 to November 2018, 622 women with lesions suspicious for breast cancer on mammography or ultrasonography (i.e., BI-RADS category 4 or 5) underwent MRI examinations with multi-b DWI. The exclusion criteria included the following: patients previously treated for a malignancy (*N* = 13), patients without histopathological results (*N* = 27), and patients with motion artifacts (*N* = 5), lesions that were not seen in DWI mappings (*N* = 25), and the mean value of the goodness-of-fit of the diffusion fitting model was less than 0.8 (*N* = 10). Ultimately, a total of 542 women (mean age, 51 years; age range, 24–84 years), with 542 lesions were enrolled in this study.

### MR imaging

All breast MRI examinations were performed on a 1.5 T MR scanner (MAGNETOM Aera, Siemens Healthcare, Erlangen, Germany) with a dedicated 18-channel phased-array breast coil. The breast MR examinations included fat-suppressed T2-weighted fast spin-echo imaging, T1-weighted imaging (T1WI), DWI, and DCE T1WI. All MR imaging examinations were performed before biopsy. The parameters of the above sequences are shown in Additional file 1: Appendix S1.

### Image postprocessing and lesion segmentation

**Fig. 1:**
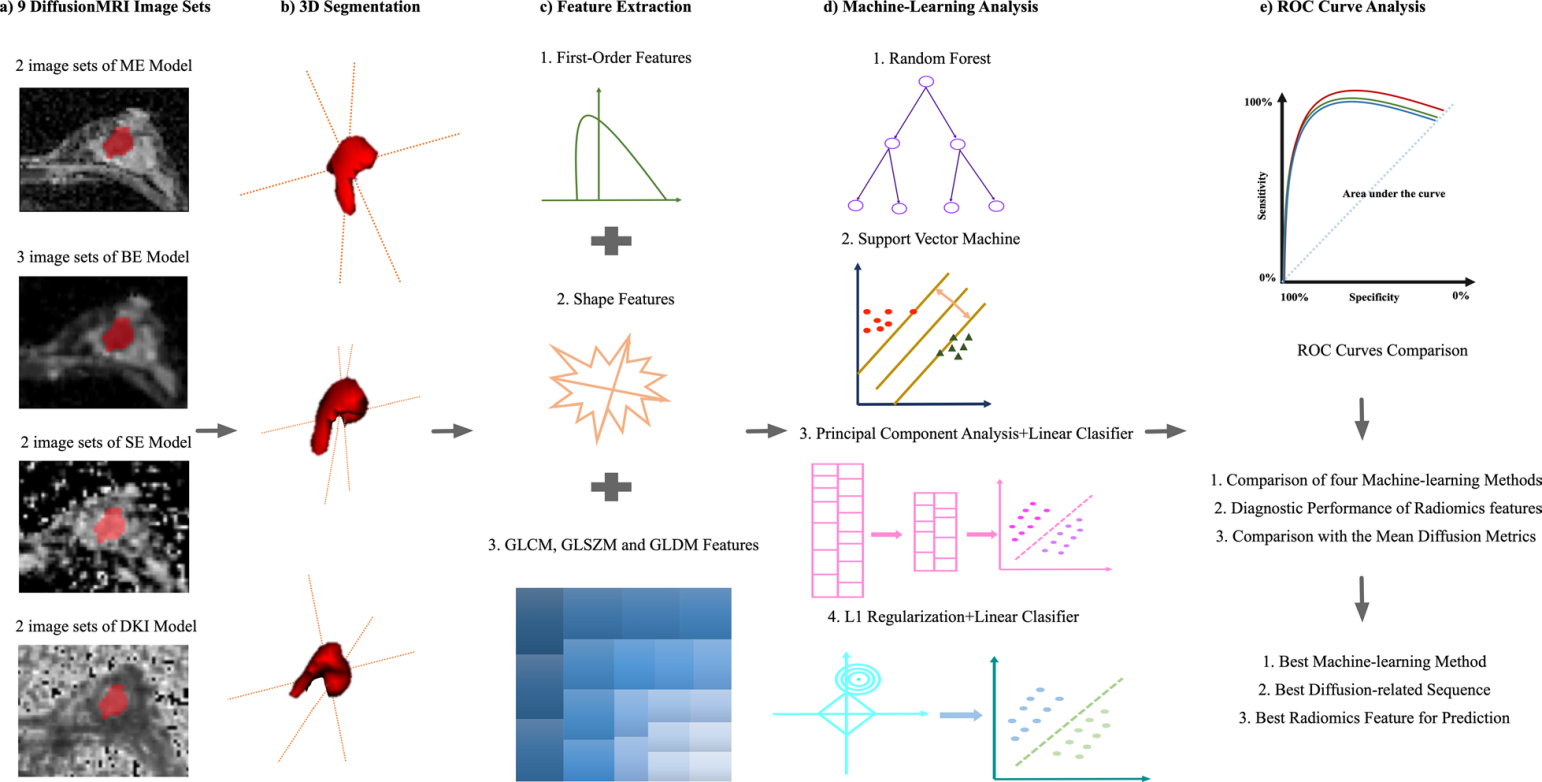
Workflow of image processing. **a** MRI data of multi-b value sequences and quantitative maps from ME, BE, SE and DKI models. **b** 3D segmentations of lesions shown as surface shaded 3D renderings. **c** Extraction of radiomics features, i.e., First-order, Shape, GLCM, GLSZM and GLDM. **d** Radiomics analysis using four models (RF, SVM, PCA-LR, and L1R-LR), and **e** ROC curve analysis. ROC curves are used for the comparison of four methods, and diagnostic performance of radiomics features and mean diffusion metrics

After data acquisition, all images were transferred to N4ITK for the data normalization. Then, all these data were assessed by KS and WC (with 8 years and 12 years of experience in breast imaging) to identify all lesions by using the DWI source images with b values of 1000 s/mm^2^, T2-weighted images, and the first phase of postcontrast T1-weighted images. Clinical information and the X-ray and US images were provided to the radiologists. The lesions were manually segmented in the DW images (b_1000_) on all visible sections, resulting in a three-dimensional image of the lesion. Lesions were segmented by using the inner border of the lesion to minimize partial volume effects. All volumes of interest (VOIs) were manually segmented and labeled via a free open-source software package (ITK-SNAP, version 3.4.0, http://www.itksnap.org). An overview of our workflow is illustrated in Fig. 1.

### Diffusion data analysis and processing

All diffusion parameter maps were generated using an in-house MATLAB software (MathWorks, Natick, MA, USA). The software first applied a Gaussian filter with a full width at a half maximum of 3 mm to suppress noise in the diffusion images before the pixel-by-pixel fitting process. Four diffusion models are described as follows:

1. ME model
  ADC maps were generated according to the following equation:
  $$S_{b} /S_{0} = exp \, \left( { - b \cdot ADC} \right),$$
  where *S*_*b*_ represents signal intensity in the presence of diffusion sensitization, and *S*_*0*_ represents signal intensity in the absence of diffusion sensitization. The ADC__all-b_ maps were generated by using all 13 b values. The ADC_0–1000_ maps were generated by using b values of 0 and 1000.
2. BE_IVIM model
  The IVIM parameters were fitted using the following IVIM model (proposed by Le Bihan et al*. *[11, 12]*:*
  $$S_{b} /S_{0} = \left( {1 - f} \right) \cdot exp\left( { - b \cdot D} \right) + f \cdot exp \, \left( { - b \cdot D^{*} } \right),$$
  where *D* is the true diffusion as reflected by the pure molecular diffusion, *f* is the fractional perfusion related to microcirculation, and D^*^ is the pseudo-diffusion coefficient that represents perfusion-related diffusion or incoherent microcirculation.
3. SE model
  The SE model was used to obtain the molecular water diffusion heterogeneity index (*α*) and the distributed diffusion coefficient (*DDC*) through the following equation:
  $$S_{b} /S_{0} = exp\left[ { - \, \left( {b \cdot DDC} \right)^{\alpha } } \right],$$
  where *α* is related to the intravoxel molecular water diffusion heterogeneity, which ranges from 0 to 1. A numerically high *α* value represents low intravoxel diffusion heterogeneity (approaching mono-exponential decay). *DDC* represents the mean intravoxel diffusion rate.
4. DKI model
  Calculation of DKI parameters was performed by fitting the following nonlinear equation:
  $$S_{b} /S_{0} = exp\left( { - b \cdot D + 1/6 \cdot b^{2} \cdot D^{2} \cdot K} \right),$$
  where *K* is a unitless parameter that quantifies the deviation of water motion from the Gaussian distribution. *K* is zero for a perfect Gaussian diffusion, and a large *K* indicates considerable deviation of diffusion from a perfect Gaussian behaviour. *D* is a corrected *ADC* by removing non-Gaussian bias.

### Feature extraction

Radiomics features were calculated using the PyRadiomics Python package (version 2.1.2), and the recommended default settings were used for the analysis [26]. Each map extracted 100 features comprising 18 first-order (FO) features, 14 shape features, 22 Gy level co-occurrence matrix (GLCM) features, 16 Gy level run length matrix (GLRLM) features, 16 Gy level size zone matrix (GLSZM) features, and 14 Gy level dependence matrix (GLDM) features. Details of the extracted features are shown in Additional file 1: Appendix S2. In total, 900 features were extracted. The interclass correlation coefficients (ICCs) were used to determine the interobserver reproducibility of the radiomics features [27].

The mean diffusion metrics of ME (mADC_all-b_, mADC_0–1000_), BE (mD, mD*, mf), SE (mDDC, mα), and DKI (mK, mD) were extracted from the radiomics set for separate analysis. Feature importance (FI) was calculated by using random forest. Feature importance was determined as the mean decrease in the impurity of the random forest as previously described [28].

### RF, L1R, PCA, and SVM

The 542 subjects were randomly and equally divided into a training set containing 271 subjects and an independent testing set containing the remaining 271 subjects. The ratios of malignant and benign subjects in the training set and the testing set were equal to the ratio in the whole dataset. The RF, SVM, PCA-LR, and L1R-LR algorithms were all based on the most widely used machine learning Python package, i.e., Scikit-learn [29]. For RF, the parameters were set as the default values, the number of trees was 100, and the maximum depth of the tree was 3. For L1 regularization (L1R), the features were selected implicitly by the L1 regularization of the linear classifier. L1R enforced the coefficients of the linear model to be sparse, thus making a small subset of radiomics features contribute to the final results. For PCA, 100 features were selected based on their power to differentiate benign from malignant lesions in the training set by sorting the lowest *P* values. Then, the first 10 principal components were chosen for the linear model for prediction. The parameter settings of both PCA and L1R followed the widely-used strategies in other MRI-based radiomics studies for breast cancer [23]. For SVM, we used the radial basis function (RBF) kernel. The parameters were optimized with respect to the training set. The hyperparameters of the above four methods are shown in Additional file 1: Appendix S3. The classifiers were trained using the repeated tenfold cross-validation (CV) method (100 times) in the training cohort, and their prognostic performance was then evaluated in the validation cohort using the area under the receiver operating characteristic (ROC) curve. A more detailed description of the frequencies of the features of RF during 100 times of tenfold CV is shown in Additional file 1: Appendix S4.

### Statistical analysis

A goodness-of fit evaluation was performed for fitting of the BE, SE and DKI models by using MATLAB (MathWorks). The R^2^ value was calculated [9]. ROC curves were generated for the mean diffusion metrics (ME-mADC_all b_, ME-mADC_0–1000_, BE-mD, BE-mD^*^, BE-mf, SE_mDDC, SE_mα, DKI-mK, and DKI-mD), and the ROC curves of all the 9 DWI image sets of the RF, L1R, PCA, and SVM models were calculated for comparison. The ROC curves of the 9 diffusion-related image sets were calculated from the results obtained by the CV models in the independent testing set. To compare the AUCs of the mean diffusion metrics and radiomics features, the McNemar test was used for the paired cases. All these comparisons were run 100 times, and we obtained the mean *P* values. Bonferroni adjustment was performed to control for α error inflation [29]. A *P* value less than 0.05/23 (0.00217) was regarded as a significant difference. All statistical evaluations were performed by using software developed either with the Python programming language [30] or with MATLAB software.

## Results

### Image quality of multi-b diffusion weighted imaging

The mean R^2^ value for the BE model fit was 0.90 ± 0.06. The mean R^2^ value for the SE model fit was 0.95 ± 0.03. The mean R^2^ value for the DKI model fit was 0.99 ± 0.01.

The signal intensity of malignant lesions on the map of b_2500_ was 113. 25 ± 31.53. The signal intensity of benign lesions on the map of b_2500_ was 36.83 ± 10.73. The signal to noise ratio (SNR) of b_2500_ was 30.01 ± 10.16. The contrast noise ratio (CNR) of b_2500_ was 2.25 ± 0.67. The lesion contrast on the map of b_2500_ was 3.20 ± 1.04. A case of 23 datasets is shown in Additional file 1: Appendix S5.

### Patient demographic characteristics

There was significant difference in demographic characteristics between patients with malignant lesions and patients with benign lesions (55.0 ± 12.2 vs. 50.3 ± 11.6, *P* < 0.001).

### Pathological features

Of the 542 lesions, 333 were malignant, and 209 were benign. The malignant lesions included ductal carcinoma in situ (*N* = 28), lobular carcinoma in situ (N = 1), invasive carcinoma (*N* = 274), invasive lobular carcinoma (*N* = 1), invasive solid papillary carcinoma (*N* = 9), malignant phyllodes tumors (*N* = 3), mucinous carcinoma (*N* = 8), metaplastic cancer (*N* = 1), diffuse large B-cell lymphoma (*N* = 2), encapsulated papillary carcinoma (*N* = 3), and invasive micropapillary carcinoma (*N* = 3). Benign lesions included fibroadenoma (N = 101), benign phyllodes tumors (*N* = 3), fibrocystic change (*N* = 4), cyst combined chronic infection (*N* = 6), papilloma (*N* = 54), usual ductal hyperplasia (*N* = 16), fat necrosis (*N* = 1), and adenosis (*N* = 24).

### Comparison of RF, L1R-LR, PCA-LR, and SVM in the diagnosis of breast lesions with multi-b diffusion-weighted imaging

The AUCs of RF in the differential diagnosis of breast lesions ranged from 0.80 (BE_D*) to 0.85 (BE_D), whereas the AUCs of PCA-LR ranged from 0.53 (SE_DDC) to 0.78 (BE_D*). The AUCs of L1R-LR and SVM ranged from 0.53 (SE_DDC) to 0.83 (ME_ADC_0–1000_) and from 0.51 (SE_DDC) to 0.81 (ME_ADC_0–1000_), respectively.

The top image image sets with the highest AUCs by the RF were BE_D (0.85), ME_ADC_all b_ (0.84), DKI_K (0.84), ME_ADC_0–1000_ (0.83) and DKI_D (0.83). The results of all AUCs by RF are shown in Table 1. The top five image sets with the highest mean AUCs were ME_ADC_0–1000_ (0.81), BE_D (0.81), ME_ADC_all b_ (0.81), DKI_D (0.80), and DKI_K (0.80).

**Table 1:** Comparisons between radiomics and mean diffusion metrics

Maps	AUC of mean metrics	AUC of Radiomics	*P* value
ME			
mADC_0–1000_	0.79 (0.76–0.83)	0.83 (0.80–0.87)	0.002
mADC_all-b_	0.77 (0.73–0.81)	0.84 (0.80–0.88)	0.001
BE_IVIM			
mD	0.75 (0.72–0.79)	0.85 (0.81–0.89)	< 0.001
mD*	0.67 (0.63–0.71)	0.80 (0.74–0.83)	< 0.001
mf	0.54 (0.50–0.58)	0.82 (0.77–0.86)	< 0.001
SE			
mDDC	0.77 (0.74–0.81)	0.81 (0.77–0.85)	0.030
mα	0.62 (0.58–0.66)	0.80 (0.77–0.84)	< 0.001
DKI			
mD	0.78 (0.73–0.80)	0.83 (0.80–0.88)	0.005
mK	0.75 (0.71–0.80)	0.84 (0.80–0.88)	0.001

ME: mono-exponential model; mADC_0–1000_: mean value of ADC_0–1000;_ mADC_all b_: mean value of ADC_all b_; BE-IVIM: biexponential intravoxel incoherent motion model; mD: mean value of true diffusion coefficient; mD*: mean value of pseudo-diffusion coefficient; mf: mean value of fractional perfusion; SE: stretched exponential model; mDDC: mean value of distributed diffusion coefficient; m*α*: mean value of low intravoxel diffusion heterogeneity; DKI: diffusion kurtosis imaging; mD: mean value of diffusivity coefficient; mK: mean value of kurtosis coefficient

Details on the top five image sets with the highest mean AUCs by RF, SVM, L1R-LR, and PCA-LR are shown in Table 2. The comparisons between RF and L1R, and between PCA and SVM are shown in Additional file 1: Appendix S6.

**Table 2:** Diagnostic performance of ME_ADC_0–1000_, BE_IVIM_D, ME_ADC_all b_, DKI-D and DKI-K by using RF, L1R-LR, PCA-LR, and SVM, respectively

Maps	AUC (95% CI)				
	RF	L1R-LR	PCA-LR	SVM	mAUC
ME-ADC_0–1000_	0.83 (0.80–0.87)	0.83 (0.79–0.87)	0.76 (0.70–0.81)	0.81 (0.76–0.85)	0.81
BE-IVIM-D	0.85 (0.81–0.89)	0.83 (0.78–0.87)	0.75 (0.70–0.82)	0.80 (0.75–0.85)	0.81
ME-ADC_all b_	0.84 (0.80–0.87)	0.82 (0.79–0.87)	0.77 (0.70–0.83)	0.79 (0.74–0.85)	0.81
DKI-D	0.83 (0.80–0.86)	0.83 (0.78–0.86)	0.75 (0.74–0.82)	0.80 (0.77–0.84)	0.80
DKI-K	0.84 (0.81–0.89)	0.83 (0.78–0.87)	0.74 (0.70–0.80)	0.79 (0.75–0.85)	0.80

RF: random forest; SVM: support vector machine; PCA: principal component analysis; L1R: L1 regularization; LR: linear regression; mAUC: mean values of AUCs of RF, L1R-LR, PCA-LR and SVM

RF achieved the highest frequency of the highest AUCs compared with L1R-LR, PCA-LR, and SVM (8/9 vs. 1/9 vs. 0/9 vs. 0/9, *P* < 0.001). The mean AUCs of the nine image sets by RF, L1R-LR, PCA-LR and SVM were 0.82, 0.78, 0.73, and 0.76, respectively.

### Diagnostic performance comparison of radiomics features by RF and the mean values of diffusion metrics

The interobserver reproducibility of radiomics feature extraction was satisfactory, with ICCs greater than 0.80 for all extracted features. The AUCs of the radiomics features for the differential diagnosis of breast lesions ranged from 0.80 (BE_D*) to 0.85 (BE_D), with a sensitivity of 83% to 88%, and a specificity of 74% to 82%. The AUCs of the mean diffusion metrics ranged from 0.54 (BE_mf) to 0.79 (ME_mADC_0–1000_), with a sensitivity of 74% to 88%, and a specificity of 41% to 71%. The AUCs of the radiomics features for the differential diagnosis of breast lesions were higher than those of the corresponding mean diffusion metrics, and there were significant differences in the AUCs between the mean values of the diffusion metrics (ME_mADC_all-b_, ME_mADC_0–1000_, BE_mD, BE_mD^*^, BE_mf, SE_mα, and DKI_mK) and the corresponding radiomics features of AUCs (all *P* < 0.002) for the differentiation of benign and malignant breast lesions. Details of the comparison are shown in Table 3.

**Table 3:** Diagnostic performance of multi-b diffusion maps based on ME, BE, SE and DKI models

Maps	AUC (95% CI)	Sensitivity% (95% CI)	Specificity% (95% CI)	PPV% (95% CI)	NPV% (95% CI)
ME					
ADC_0–1000_	0.83 (0.80–0.87)	88 (82–93)	79 (68–87)	87 (81–92)	80 (72–88)
ADC_all-b_	0.84 (0.80–0.88)	88 (82–93)	81 (71–90)	88 (82–94)	80 (72–88)
BE-IVIM					
D	0.85 (0.81–0.89)	88 (81–93)	82 (73–90)	89 (83–93)	80 (72–89)
D*	0.80 (0.74–0.83)	83 (76–88)	77 (66–86)	85 (77–91)	73 (65–81)
f	0.82 (0.77–0.86)	85 (79–90)	79 (68–87)	86 (80–92)	77 (68–84)
SE					
DDC	0.81 (0.77–0.85)	85 (78–90)	77 (66–86)	85 (79–91)	76 (68–84)
α	0.80 (0.77–0.84)	85 (79–89)	76 (67–86)	85 (80–91)	76 (69–82)
DKI					
D	0.83 (0.80–0.88)	87 (80–94)	79 (69–88)	87 (81–93)	80 (71–89)
K	0.84 (0.80–0.88)	87 (80–92)	81 (72–89)	88 (83–93)	79 (72–86)

*AUC* area under the receiver operating characteristic curve, *CI* confidence interval, *PPV* positive predictive value, *NPV* negative predictive value

### Importance of diffusion-related radiomics features

Details of all radiomics feature importance were shown in Additional file 1: Appendix S7. Regarding the radiomics features computed from nine image sets, the top five important features were FO-10 percentile (FI = 0.043), FO-Median (FI = 0.030), Shape-Sphericity (FI = 0.030), FO-Skewness (FI = 0.029), and Shape-Flatness (FI = 0.026).

**Fig. 2:**
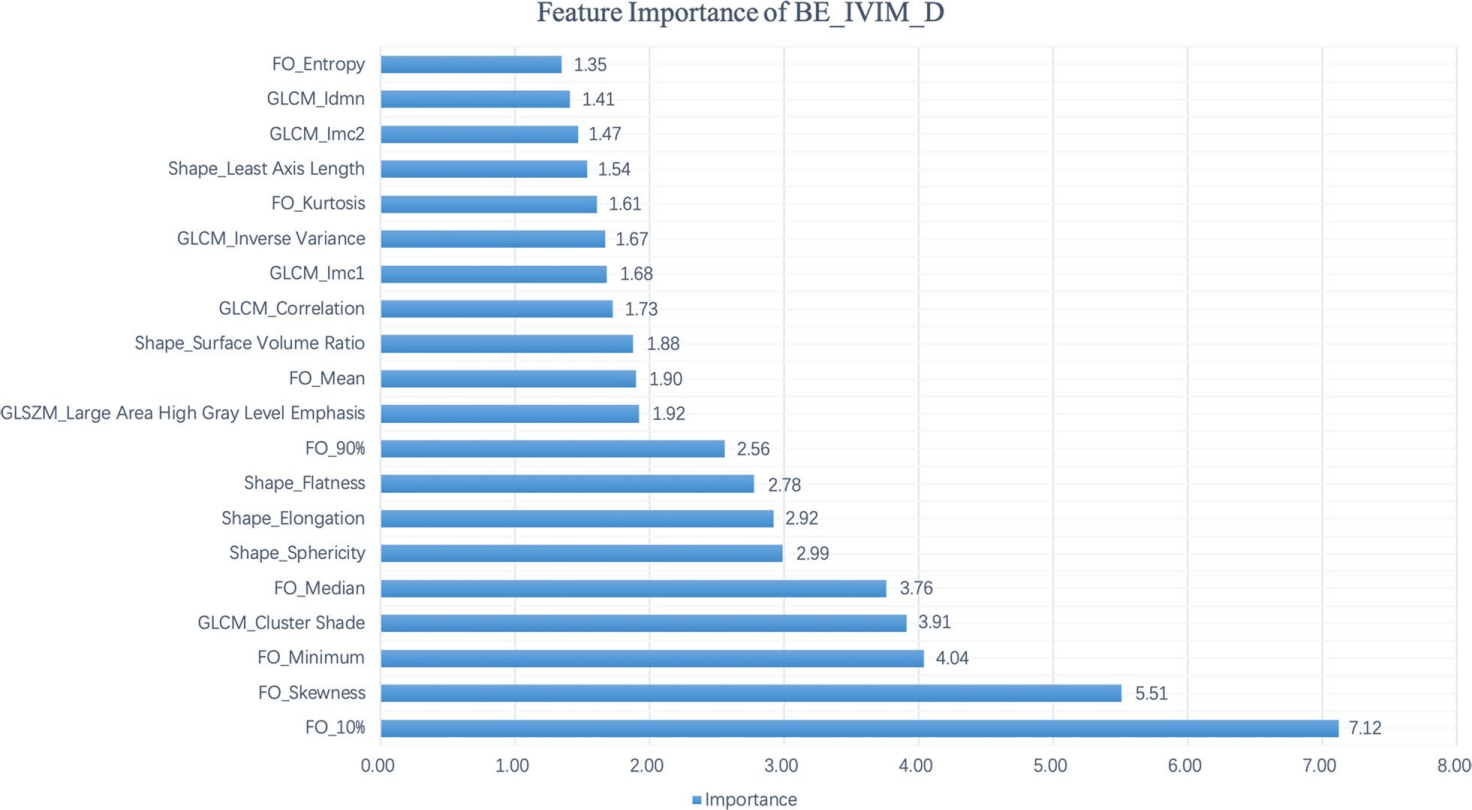
Top 20 radiomics features of BE_IVIM_D, ranked by the mean decrease in impurity of RF

**Fig. 3:**
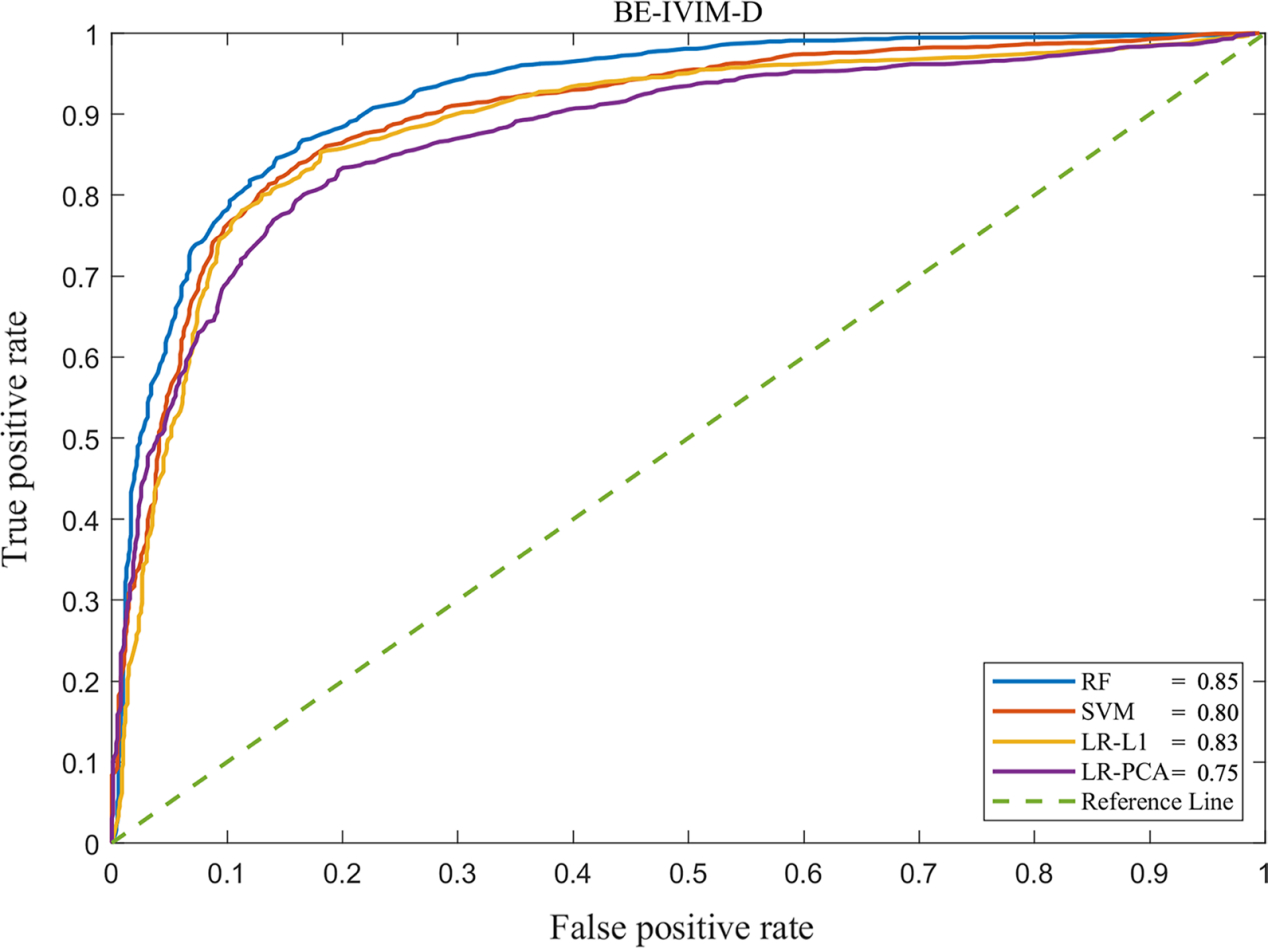
ROC curve analysis of BE_IVIM_D for radiomics-based analysis with RF, L1R, PCA, and SVM, respectively

Of the radiomics features computed from the map of BE_IVIM_D, which had the highest AUC (0.85), the top five most important features were FO-10 percentile (FI = 0.07), FO-Skewness (FI = 0.06), FO-Minimum (FI = 0.04), GLCM-Cluster Shade (FI = 0.04), and FO-Median (FI = 0.02). Details of the top 20 important features of BE_IVIM_D are shown in Fig. 2. The ROC curve of BE_IVIM_D is shown in Fig. 3.

## Discussion

Based on experimental results of this study, the BE_IVIM_D map (with the highest AUC by RF), and the FO-10 percentile feature (with the highest FI by RF) from the radiomics-based analysis of multiparametric DWI are recommended in the characterization of breast lesions. Furthermore, we also found that the diagnostic performance of multiparametric DWI-derived radiomics was superior to that of the mean diffusion metrics in differentiating between benign and malignant breast lesions. This finding suggests that the radiomics-based analysis for multiparametric DWI has a potentially-improved performance in the classifications of breast lesions.

The majority of radiomics-based analyses in breast MRI research utilize T2WI, contrast T1WI, and conventional DWI [17, 18, 31, 32]. To the best of our knowledge, this is the first study that extensively explored radiomics from multi-b-value maps and its commonly used fitting models (ME, BE, SE, and DKI), which could reflect more details of both Gaussian and non-Gaussian water diffusion distributions in tumors. Bickelhaupt et al. [17] demonstrated that the radiomics features of DKI can help differentiate malignant breast lesions from benign lesions. However, they only used the fitting model of DKI, and their scan sequences contained both the single-shot echo planar imaging (ss-EPI) in 95 patients and readout-segmented echo-planar imaging (rs-EPI) in 127 patients. We used four clinically used diffusion fitting models, and we also enlarged the sample size (542 lesions) in our study. Moreover, all the patients in our study were scanned with rs-EPI, which has significantly higher image quality and lesion conspicuity than ss-EPI, as suggested by previous studies [33, 34].

Many radiomics-based machine learning methods can be used for lesion classification [17, 18, 35, 36]. In this study, we extensively explored four promising algorithms of RF, L1R-LR, PCA-LR and SVM, which have been demonstrated to have high effectiveness in the previous radiomics studies [23, 37]. We found that the ADC_0–1000_ feature attained the highest mean AUC with all four algorithms, indicating that the mono-exponential model had already provided enough diagnostic information for breast cancer. Furthermore, RF had the highest probability of achieving the highest AUCs (8/9). Accordingly, this finding further corroborates the robustness and strong generalization power of RF [28]. Thus, in our further analysis, both the calculation of feature importance and the comparison of AUCs were based on the results of RF.

The most predictive image set by RF (i.e., with the highest AUC, sensitivity and specificity) was BE_IVIM_D. Of note, BE_IVIM_D can remove the influence of perfusion and therefore reflects the true diffusion coefficient, better reflecting water movement in the living tissues. This may be the reason why the radiomics features computed from BE_IVIM_D provide more accurate information on water diffusion in breast cancer classification. Furthermore, the most important radiomics feature of BE_IVIM_D is the FO-10-percentile. Unlike that in previously reported studies [38–40], our experimental results did not suggest that texture features can attain better performance than the FO features on BE_IVIM_D. Accordingly, the FO features may be more predictive of lesion malignancy. On the other hand, the FO-10-percentile was also shown to be the most important feature (FI = 0.043) for the differentiation of benign and malignant breast lesions, indicating that first-order features remain important cues in multiparametric DWI for the differential diagnosis of breast lesions.

Our study has several limitations. First, all lesions in this study were drawn manually, which was time-consuming. Thus, automated lesion segmentation will be implemented in our future study to improve the objectiveness of lesion boundaries and to expedite preprocessing. Second, our multi-b value sequences were acquired with a fixed protocol, whereas the choice of optimal b-values could vary across different institutions. Thus, there was a lack of an external independent verification dataset to verify the generalization ability of this study’s findings. Finally, this study employed all extracted diffusion-related radiomics for breast cancer diagnosis. The feature selection strategy was not implemented in this study. In future studies, we will conduct feature selection to optimize the construction of radiomics models.

## Conclusions

In conclusion, the BE_IVIM_D map, and of FO-10-percentile feature by RF enabled accurate differentiation between malignant and benign breast lesions. Radiomics features computed from multiparametric DWI performed better than the mean values in distinguishing benign and malignant breast lesions. Hence, our study may shed a light on the applicability of radiomics from the multiparametric DWI for the clinical diagnosis of breast lesions.

## Supplementary Information

**Additional file 1.** The scanning parameters of T2WI, multi-b DWI, pre-contrast T1WI, and DCE T1WI.

## Data Availability

The datasets used or analyzed during the current study are available from the corresponding author on reasonable request.

## Abbreviations

DWI: Diffusion weighted imaging
ADC: Apparent diffusion coefficient
ROC: Receiver operating characteristic
AUC: Area under the ROC curve
BI-RADS: Breast imaging reporting and data system
Ss-EPI: Single-shot echo planar imaging
RS-EPI: Readout segmented echo planar imaging
ME: Mono exponential model
ME_ADC_0__–1000_: ADC_0__–1000_ map of ME model
mADC_0__–1000_: Mean value of ADC_0__–1000_
BE: Bi exponential model
D*: Pseudo-diffusion coefficient
D: True diffusion coefficient
f: Fractional perfusion
BE_D*: D* map of BE model
mD*: Mean value of D*
SE: Stretched exponential model
DDC: Distributed diffusion coefficient
*α*: Low intravoxel diffusion heterogeneity
SE_DDC: DDC map of SE model
mDDC: Mean values of DDC
CI: Confidence interval
DKI: Diffusion kurtosis imaging
K: Kurtosis coefficient
D: Diffusivity coefficient
DKI_K: Kurtosis map based on DKI model
DKI_D: Diffusivity map based on DKI model
mK: Mean value of kurtosis coefficient
mD: Mean value of diffusivity coefficient
IVIM: Intravoxel incoherent motion
RF: Random forest
SVM: Support vector machine
PCA: Principal component analysis
L1R: L1 regularization
LR: Linear regression
FO: First order
DCE: Dynamic contrast-enhancement
T2WI: T2-weighted imaging
T1WI: T1-weighted imaging
IRB: Institutional review board
GLCM: Gray level co-occurrence matrix
GLRLM: Gray level run length matrix
GLSZM: Gray level size zone matrix
GLDM: Gray level dependence matrix
ICC: Interclass correlation coefficient
RBF: Radial basis function
CV: Cross validation
SNR: Signal to noise ratio
CNR: Contrast noise ratio
FI: Feature importance
PPV: Positive predictive value
NPV: Negative predictive value
