# Supplementary material for: Radiomics-based machine learning analysis and characterization of breast lesions with multiparametric diffusion-weighted MR
Source: J Transl Med. 2021 Oct 24;19:443. doi: 10.1186/s12967-021-03117-5 (PMC8543912; doi:10.1186/s12967-021-03117-5)

**Appendix S1**

The T2-weighted fast spin-echo imaging was acquired with the following parameters: TR/TE of 4990/65 ms, field of view of 360 × 360 mm^2^, slice thickness of 4.0 mm, acquisition time of 2 mins and 11 seconds. Multi-b DWI was acquired by using readout-segmented echo-planar imaging (rs-EPI) with the following parameters: TR/TE of 5500/80, slice of 20, flip angle of 180 degree, readout segments of 3, slice thickness of 5 mm, field of view of 340 × 340 mm^2^, PAT mode of GRAPPA, matrix of 172 ×172, fat saturation mode of spectral adiabatic inversion recovery (SPAIR), b values of 0, 10, 20, 30, 40, 50, 100, 200, 400, 800, 1000, 1500, 2000, 2500, acquisition time of 10 mins and 29 seconds; signal to noise ratio of 1. DCE was obtained using a fat-suppressed T1-weighted gradient-echo sequence before, and four times continuously after injection of a gadolinium contrast medium (Magnevist, Bayer HealthCare Pharmaceuticals INC., Wayne, New Jersey, USA), which was administered intravenously by a power injector at a dose of 0.1mmol/kg of body weight at a rate of 2.5 mL/s, followed by a 20-mL saline flush at the same injection rate. The scan parameters of DCE were as follows: TR/TE of 4.5/1.6 ms, field of view of 340 × 340 mm^2^, matrix of 384 × 385, flip angle of 10 degree, slice thickness of 1.0 mm, number of slices 104, and the total duration of T1-weighted imaging of 7 mins and 30 seconds.

**Appendix S2 Detailed information about extracted radiomics features**

Extracted 100 image features were divided into six groups:

(1) **18 First-order (FO) features**: 10 percentile, 90 percentile, Energy, Entropy, Interquartile Range, Kurtosis, Maximum, Mean Absolute Deviation, Mean, Median, Minimum, Range, Robust Mean Absolute Deviation, Root Mean Squared, Skewness, Total Energy, Uniformity, and Variance.

(2) **14 Shape features**: Elongation, Flatness, Least Axis Length, Major Axis Length, Maximum 2D Diameter Column, Maximum 2D Diameter Row, Maximum 2D Diameter Slice, Maximum 3D Diameter, Mesh Volume, Minor Axis Length, Sphericity, Surface Area, Surface Volume Ratio, and Voxel Volume.

(3) **22 Gray level co-occurrence matrix (GLCM) features**: Autocorrelation, Joint Average, Cluster Prominence, Cluster Shade, Cluster Tendency, Contrast, Correlation, Difference Average, Difference Entropy, Difference Variance, Joint Energy, Joint Entropy, Imc1, Imc2, Idm, Idmn, Id, Idn, Inverse Variance, Maximum Probability, Sum Entropy, and Sum Squares.

(4) **16 Gray level run length matrix (GLRLM) features**: Gray Level Non Uniformity, Gray Level Variance, Gray Level Non Uniformity Normalized, High Gray Level Run Emphasis, Long Run Emphasis, Long Run High Gray Level Emphasis, Long Run Low Gray Level Emphasis, Low Gray Level Run Emphasis, Run Entropy, Run Length Non Uniformity, Run Length Non Uniformity Normalized, Run Percentage, Run Variance, Short Run Emphasis Short Run High Gray Level Emphasis, and Short Run Low Gray Level Emphasis.

(5) **16 Gray level size zone matrix (GLSZM) features**: Gray Level Non Uniformity, Gray Level Non Uniformity Normalized, Gray Level Variance, High Gray Level Zone Emphasis, Large Area Emphasis, Large Area High Gray Level Emphasis, Large Area Low Gray Level Emphasis, Low Gray Level Zone Emphasis, Size Zone Non Uniformity, Size Zone Non Uniformity Normalized, Small Area Emphasis, Small Area High Gray Level Emphasis, Small Area Low Gray Level Emphasis, Zone Entropy, Zone Percentage, and Zone Variance.

(6) **14 Gray level dependence matrix (GLDM) features**: Dependence Entropy, Dependence Non Uniformity, Dependence Non Uniformity Normalized, Dependence Variance, Gray Level Non Uniformity, Gray Level Variance, Gray Level Non Uniformity, Gray Level Variance, High Gray Level Emphasis, Large Dependence Emphasis, Large Dependence High Gray Level Emphasis, Large Dependence Low Gray Level Emphasis, Low Gray Level Emphasis, Small Dependence Emphasis, Small Dependence High Gray Level Emphasis, and Small Dependence Low Gray Level Emphasis. Details about the features’ calculation are in the website: <https://pyradiomics.readthedocs.io/en/latest/features.html>

**Appendix S3 Hyperparameters of four methods**

| Methods | Hyperparameters |
| --- | --- |
| L1R | C= 1 |
| PCA | N_Components = 10  Solver = svd_solver |
| SVM | C = 1  Kernel = 'rbf'  Gamma = 'auto'  Shrinking = True  Probability = False  Tol = 1e3  Class_weight = 'balanced'  Decision_function_shape = 'ovr' |
| RF | N_trees = 100  Base_score = 0.5  Booster = 'gbtree'  Colsample_bylevel = 1  Colsample_bynode = 1  Colsample_bytree = 1  Gamma = 0  Learning_rate = 0.1  Max_delta_step = 1  Max_depth = 3  Min_child_weight = 1  Missing = None  N_estimators = 100  N_jobs = 1  Nthread = None  Objective = 'binary:logistic'  Random_state = 0  Reg_alpha = 0  Reg_lambda = 1  Scale_pos_weight = 'balanced' |

**Appendix S4 Frequency of Top 100 Features in maps with the Top 5 highest AUCs during 10-fold CV**

| Frequency | Name of Feature | Kind of Feature | Map |
| --- | --- | --- | --- |
| 1000/1000 | Variance | FO | BE_IVIM_D |
| 994/1000 | Zone Entropy | GLSZM | b_2500_ |
| 990/1000 | Robust Mean Absolute Deviation | FO | BE_IVIM_D |
| 988/1000 | Kurtosis | FO | DKI_K |
| 976/1000 | Energy | FO | BE_IVIM_D |
| 974/1000 | Range | FO | BE_IVIM_D |
| 974/1000 | Gray Level Non-Uniformity Normalized | GLRLM | DKI_K |
| 958/1000 | Dependence Non-Uniformity | GLDM | BE_IVIM_D |
| 954/1000 | Small Dependence Low Gray Level Emphasis | GLDM | SE_DDC |
| 942/1000 | Dependence Variance | GLDM | SE_DDC |
| 929/1000 | Contrast | GLCM | DKI_K |
| 920/1000 | Correlation | GLCM | DKI_K |
| 918/1000 | Long Run Emphasis | GLRLM | b_2500_ |
| 907/1000 | Long Run Low Gray Level Emphasis | GLRLM | b_2500_ |
| 907/1000 | Gray Level Variance | GLDM | SE_DDC |
| 887/1000 | Maximum Probability | GLCM | DKI_K |
| 877/1000 | 90 Percentile | FO | ME_ADC _all_b_ |
| 873/1000 | Large Area Low Gray Level Emphasis | GLSZM | BE_IVIM_D |
| 854/1000 | Gray Level Non-Uniformity | GLDM | SE_DDC |
| 852/1000 | Short Run High Gray Level Emphasis | GLRLM | DKI_K |
| 844/1000 | Entropy | FO | BE_IVIM_D |
| 843/1000 | Gray Level Variance | GLDM | BE_IVIM_D |
| 829/1000 | Surface Volume Ratio | Shape | All |
| 811/1000 | Low Gray Level Emphasis | GLDM | SE_DDC |
| 810/1000 | Sum Entropy | GLCM | ME_ADC _all_b_ |
| 809/1000 | Joint Energy | GLCM | DKI_K |
| 800/1000 | Joint Energy | GLCM | BE_IVIM_D |
| 799/1000 | Gray Level Variance | GLSZM | b_2500_ |
| 793/1000 | Contrast | GLCM | b_2500_ |
| 786/1000 | Inverse Variance | GLCM | b_2500_ |
| 775/1000 | Variance | FO | b_2500_ |
| 773/1000 | Interquartile Range | FO | DKI_K |
| 760/1000 | Large Dependence Low Gray Level Emphasis | GLDM | BE_IVIM_D |
| 748/1000 | Imc1 | GLCM | b_2500_ |
| 742/1000 | Total Energy | FO | ME_ADC _all_b_ |
| 726/1000 | Energy | FO | SE_DDC |
| 716/1000 | Contrast | GLCM | BE_IVIM_D |
| 713/1000 | Difference Entropy | GLCM | b_2500_ |
| 707/1000 | Joint Energy | GLCM | b_2500_ |
| 692/1000 | Sphericity | Shape | All |
| 692/1000 | Mean Absolute Deviation | FO | DKI_K |
| 691/1000 | Joint Entropy | GLCM | b_2500_ |
| 689/1000 | Difference Variance | GLCM | b_2500_ |
| 670/1000 | Joint Average | GLCM | DKI_K |
| 648/1000 | Robust Mean Absolute Deviation | FO | b_2500_ |
| 631/1000 | Cluster Shade | GLCM | b_2500_ |
| 628/1000 | Mean Absolute Deviation | FO | b_2500_ |
| 627/1000 | Energy | FO | ME_ADC _all_b_ |
| 615/1000 | Short Run Emphasis | GLRLM | b_2500_ |
| 613/1000 | Run Entropy | GLRLM | b_2500_ |
| 612/1000 | Idmn | GLCM | b_2500_ |
| 609/1000 | Run Variance | GLRLM | BE_IVIM_D |
| 597/1000 | Skewness | FO | b_2500_ |
| 577/1000 | Skewness | FO | ME_ADC _all_b_ |
| 571/1000 | Size Zone Non-Uniformity | GLSZM | BE_IVIM_D |
| 564/1000 | Gray Level Variance | GLSZM | SE_DDC |
| 562/1000 | Run Length Non-Uniformity Normalized | GLRLM | BE_IVIM_D |
| 558/1000 | Kurtosis | FO | BE_IVIM_D |
| 553/1000 | Mean | FO | b_2500_ |
| 547/1000 | Small Dependence Emphasis | GLDM | b_2500_ |
| 538/1000 | Kurtosis | FO | b_2500_ |
| 536/1000 | Short Run High Gray Level Emphasis | GLRLM | BE_IVIM_D |
| 514/1000 | Idn | GLCM | BE_IVIM_D |
| 510/1000 | Maximum Probability | GLCM | b_2500_ |
| 507/1000 | Cluster Prominence | GLCM | ME_ADC _all_b_ |
| 505/1000 | Small Dependence Emphasis | GLDM | BE_IVIM_D |
| 501/1000 | Low Gray Level Emphasis | GLDM | b_2500_ |
| 499/1000 | Low Gray Level Run Emphasis | GLRLM | DKI_K |
| 490/1000 | Large Dependence High Gray Level Emphasis | GLDM | SE_DDC |
| 489/1000 | Difference Average | GLCM | BE_IVIM_D |
| 475/1000 | Short Run Low Gray Level Emphasis | GLRLM | b_2500_ |
| 474/1000 | Difference Variance | GLCM | BE_IVIM_D |
| 474/1000 | Mean | FO | SE_DDC |
| 465/1000 | Joint Energy | GLCM | ME_ADC _all_b_ |
| 460/1000 | Large Dependence High Gray Level Emphasis | GLDM | b_2500_ |
| 459/1000 | Range | FO | DKI_K |
| 454/1000 | Robust Mean Absolute Deviation | FO | DKI_K |
| 449/1000 | Low Gray Level Run Emphasis | GLRLM | b_2500_ |
| 448/1000 | Idmn | GLCM | BE_IVIM_D |
| 434/1000 | Idn | GLCM | b_2500_ |
| 431/1000 | Short Run Low Gray Level Emphasis | GLRLM | DKI_K |
| 429/1000 | Elongation | Shape | All |
| 425/1000 | Run Variance | GLRLM | b_2500_ |
| 420/1000 | Small Dependence Emphasis | GLDM | ME_ADC _all_b_ |
| 413/1000 | Minimum | FO | ME_ADC _all_b_ |
| 404/1000 | Difference Entropy | GLCM | ME_ADC _all_b_ |
| 402/1000 | Short Run Emphasis | GLRLM | DKI_K |
| 394/1000 | Minimum | FO | BE_IVIM_D |
| 393/1000 | Difference Average | GLCM | b_2500_ |
| 388/1000 | Short Run High Gray Level Emphasis | GLRLM | b_2500_ |
| 386/1000 | Idm | GLCM | DKI_K |
| 384/1000 | Total Energy | FO | BE_IVIM_D |
| 378/1000 | Gray Level Non-Uniformity Normalized | GLSZM | b_2500_ |
| 376/1000 | Difference Average | GLCM | DKI_K |
| 374/1000 | Variance | FO | DKI_K |
| 373/1000 | Dependence Entropy | GLDM | DKI_K |
| 370/1000 | Low Gray Level Zone Emphasis | GLSZM | b_2500_ |
| 370/1000 | Short Run Low Gray Level Emphasis | GLRLM | BE_IVIM_D |
| 368/1000 | Small Area Emphasis | GLSZM | BE_IVIM_D |
| 367/1000 | Kurtosis | FO | ME_ADC _all_b_ |
| 365/1000 | Flatness | Shape | All |
| 365/1000 | Difference Entropy | GLCM | BE_IVIM_D |
| 349/1000 | Gray Level Non-Uniformity | GLRLM | b_2500_ |
| 334/1000 | Minimum | FO | b_2500_ |
| 332/1000 | Gray Level Variance | GLSZM | DKI_K |
| 325/1000 | Cluster Shade | GLCM | BE_IVIM_D |
| 323/1000 | Correlation | GLCM | SE_DDC |
| 320/1000 | Long Run Low Gray Level Emphasis | GLRLM | ME_ADC _all_b_ |
| 318/1000 | Contrast | GLCM | ME_ADC _all_b_ |
| 315/1000 | High Gray Level Zone Emphasis | GLSZM | BE_IVIM_D |
| 315/1000 | Imc2 | GLCM | DKI_K |
| 313/1000 | Difference Average | GLCM | ME_ADC _all_b_ |
| 310/1000 | Gray Level Non-Uniformity Normalized | GLSZM | BE_IVIM_D |
| 309/1000 | Size Zone Non-Uniformity | GLSZM | b_2500_ |
| 306/1000 | 90 Percentile | FO | BE_IVIM_D |
| 305/1000 | Total Energy | FO | DKI_K |
| 305/1000 | Idn | GLCM | DKI_K |
| 302/1000 | Small Dependence Low Gray Level Emphasis | GLDM | b_2500_ |
| 302/1000 | Imc1 | GLCM | ME_ADC _all_b_ |
| 298/1000 | Median | FO | BE_IVIM_D |
| 290/1000 | Large Dependence Low Gray Level Emphasis | GLDM | b_2500_ |
| 290/1000 | Small Dependence High Gray Level Emphasis | GLDM | SE_DDC |
| 286/1000 | Small Area Low Gray Level Emphasis | GLSZM | b_2500_ |
| 282/1000 | Interquartile Range | FO | SE_DDC |
| 277/1000 | Minor Axis Length | Shape | All |
| 277/1000 | Idm | GLCM | b_2500_ |
| 276/1000 | Small Dependence Low Gray Level Emphasis | GLDM | BE_IVIM_D |
| 275/1000 | Idm | GLCM | ME_ADC _all_b_ |
| 275/1000 | Zone Percentage | GLSZM | ME_ADC _all_b_ |
| 275/1000 | Zone Percentage | GLSZM | BE_IVIM_D |
| 273/1000 | Zone Percentage | GLSZM | b_2500_ |
| 271/1000 | Root Mean Squared | FO | DKI_K |
| 270/1000 | Cluster Shade | GLCM | DKI_K |
| 267/1000 | Run Length Non-Uniformity Normalized | GLRLM | b_2500_ |
| 267/1000 | Small Area Emphasis | GLSZM | b_2500_ |
| 265/1000 | Joint Entropy | GLCM | SE_DDC |
| 261/1000 | Joint Energy | GLCM | SE_DDC |
| 258/1000 | Imc1 | GLCM | SE_DDC |
| 257/1000 | Range | FO | b_2500_ |
| 256/1000 | Maximum Probability | GLCM | ME_ADC _all_b_ |
| 256/1000 | Autocorrelation | GLCM | DKI_K |
| 251/1000 | Large Area Emphasis | GLSZM | DKI_K |
| 250/1000 | Imc1 | GLCM | BE_IVIM_D |
| 248/1000 | 10 Percentile | FO | b_2500_ |
| 240/1000 | Small Area High Gray Level Emphasis | GLSZM | ME_ADC _all_b_ |
| 236/1000 | Idmn | GLCM | ME_ADC _all_b_ |
| 235/1000 | Large Area High Gray Level Emphasis | GLSZM | b_2500_ |
| 235/1000 | Run Entropy | GLRLM | DKI_K |
| 234/1000 | Dependence Entropy | GLDM | BE_IVIM_D |
| 233/1000 | Dependence Non-Uniformity Normalized | GLDM | b_2500_ |
| 231/1000 | Low Gray Level Zone Emphasis | GLSZM | BE_IVIM_D |
| 227/1000 | Long Run Low Gray Level Emphasis | GLRLM | DKI_K |
| 224/1000 | Idn | GLCM | ME_ADC _all_b_ |
| 224/1000 | Long Run Emphasis | GLRLM | DKI_K |
| 222/1000 | Maximum Probability | GLCM | BE_IVIM_D |
| 222/1000 | Energy | FO | DKI_K |
| 219/1000 | Zone Entropy | GLSZM | ME_ADC _all_b_ |
| 219/1000 | Short Run Emphasis | GLRLM | BE_IVIM_D |
| 217/1000 | Cluster Tendency | GLCM | b_2500_ |
| 217/1000 | Sum Squares | GLCM | b_2500_ |
| 210/1000 | Skewness | FO | SE_DDC |
| 209/1000 | Low Gray Level Emphasis | GLDM | BE_IVIM_D |
| 205/1000 | Large Area Emphasis | GLSZM | b_2500_ |
| 205/1000 | Size Zone Non-Uniformity Normalized | GLSZM | b_2500_ |
| 205/1000 | Dependence Non-Uniformity Normalized | GLDM | DKI_K |
| 194/1000 | Large Dependence Emphasis | GLDM | b_2500_ |
| 192/1000 | Autocorrelation | GLCM | b_2500_ |
| 187/1000 | Size Zone Non-Uniformity | GLSZM | ME_ADC _all_b_ |
| 187/1000 | Low Gray Level Run Emphasis | GLRLM | BE_IVIM_D |
| 187/1000 | High Gray Level Emphasis | GLDM | BE_IVIM_D |
| 185/1000 | Small Area Low Gray Level Emphasis | GLSZM | ME_ADC _all_b_ |
| 184/1000 | Maximum | FO | BE_IVIM_D |
| 182/1000 | Gray Level Variance | GLRLM | BE_IVIM_D |
| 176/1000 | Small Dependence Emphasis | GLDM | SE_DDC |
| 175/1000 | Maximum 2D Diameter Slice | Shape | All |
| 174/1000 | Correlation | GLCM | b_2500_ |
| 174/1000 | Dependence Variance | GLDM | b_2500_ |
| 173/1000 | Joint Entropy | GLCM | ME_ADC _all_b_ |
| 173/1000 | Cluster Prominence | GLCM | BE_IVIM_D |
| 169/1000 | Gray Level Variance | GLDM | ME_ADC _all_b_ |
| 167/1000 | Median | FO | ME_ADC _all_b_ |
| 167/1000 | Gray Level Non-Uniformity | GLRLM | ME_ADC _all_b_ |
| 166/1000 | 90 Percentile | FO | b_2500_ |
| 166/1000 | Short Run High Gray Level Emphasis | GLRLM | SE_DDC |
| 165/1000 | Sum Entropy | GLCM | SE_DDC |
| 165/1000 | Difference Variance | GLCM | DKI_K |
| 164/1000 | Run Entropy | GLRLM | ME_ADC _all_b_ |
| 164/1000 | Root Mean Squared | FO | BE_IVIM_D |
| 164/1000 | Correlation | GLCM | BE_IVIM_D |
| 164/1000 | Long Run High Gray Level Emphasis | GLRLM | SE_DDC |
| 158/1000 | Large Area High Gray Level Emphasis | GLSZM | BE_IVIM_D |
| 158/1000 | Sum Entropy | GLCM | DKI_K |
| 157/1000 | Zone Entropy | GLSZM | BE_IVIM_D |
| 156/1000 | Sum Squares | GLCM | ME_ADC _all_b_ |
| 155/1000 | Long Run High Gray Level Emphasis | GLRLM | b_2500_ |
| 147/1000 | Maximum2DDiameterRow | Shape | All |
| 147/1000 | Small Area Emphasis | GLSZM | DKI_K |
| 146/1000 | Major Axis Length | Shape | All |
| 146/1000 | Maximum 2D Diameter Column | Shape | All |
| 146/1000 | High Gray Level Zone Emphasis | GLSZM | DKI_K |
| 143/1000 | Mean | FO | BE_IVIM_D |
| 142/1000 | Large Area Emphasis | GLSZM | ME_ADC _all_b_ |
| 140/1000 | Run Percentage | GLRLM | b_2500_ |
| 140/1000 | Large Area Low Gray Level Emphasis | GLSZM | ME_ADC _all_b_ |
| 140/1000 | Mean Absolute Deviation | FO | BE_IVIM_D |
| 139/1000 | Uniformity | FO | b_2500_ |
| 139/1000 | Maximum | FO | SE_DDC |
| 138/1000 | Small Area High Gray Level Emphasis | GLSZM | b_2500_ |
| 138/1000 | Idm | GLCM | BE_IVIM_D |
| 137/1000 | Least Axis Length | Shape | All |
| 136/1000 | Entropy | FO | b_2500_ |
| 136/1000 | Joint Average | GLCM | ME_ADC _all_b_ |
| 134/1000 | Gray Level Non-Uniformity | GLSZM | b_2500_ |
| 134/1000 | Large Area Emphasis | GLSZM | BE_IVIM_D |
| 134/1000 | Cluster Tendency | GLCM | DKI_K |
| 132/1000 | Gray Level Non-Uniformity | GLDM | BE_IVIM_D |
| 129/1000 | Maximum | FO | b_2500_ |
| 129/1000 | Short Run Low Gray Level Emphasis | GLRLM | ME_ADC _all_b_ |
| 128/1000 | Root Mean Squared | FO | ME_ADC _all_b_ |
| 128/1000 | Sum Entropy | GLCM | BE_IVIM_D |
| 128/1000 | Kurtosis | FO | SE_DDC |
| 128/1000 | Zone Entropy | GLSZM | SE_DDC |
| 125/1000 | Long Run Low Gray Level Emphasis | GLRLM | BE_IVIM_D |
| 125/1000 | Entropy | FO | DKI_K |
| 124/1000 | Maximum | FO | ME_ADC _all_b_ |
| 123/1000 | Interquartile Range | FO | BE_IVIM_D |
| 123/1000 | Long Run High Gray Level Emphasis | GLRLM | BE_IVIM_D |
| 121/1000 | Interquartile Range | FO | b_2500_ |
| 121/1000 | Large Area Low Gray Level Emphasis | GLSZM | b_2500_ |
| 121/1000 | Median | FO | SE_DDC |
| 120/1000 | Cluster Prominence | GLCM | SE_DDC |
| 117/1000 | Mean Absolute Deviation | FO | ME_ADC _all_b_ |
| 117/1000 | Id | GLCM | ME_ADC _all_b_ |
| 116/1000 | Root Mean Squared | FO | b_2500_ |
| 116/1000 | Gray Level Non-Uniformity Normalized | GLRLM | BE_IVIM_D |
| 113/1000 | Range | FO | ME_ADC _all_b_ |
| 112/1000 | Contrast | GLCM | SE_DDC |
| 110/1000 | Gray Level Non-Uniformity | GLSZM | DKI_K |
| 107/1000 | High Gray Level Zone Emphasis | GLSZM | ME_ADC _all_b_ |
| 107/1000 | Cluster Prominence | GLCM | DKI_K |
| 105/1000 | Mean | FO | ME_ADC _all_b_ |
| 104/1000 | Imc2 | GLCM | ME_ADC _all_b_ |
| 104/1000 | Difference Variance | GLCM | SE_DDC |
| 103/1000 | Dependence Variance | GLDM | BE_IVIM_D |
| 103/1000 | Skewness | FO | DKI_K |
| 101/1000 | Interquartile Range | FO | ME_ADC _all_b_ |
| 101/1000 | Entropy | FO | SE_DDC |
| 101/1000 | Idmn | GLCM | DKI_K |
| 100/1000 | Variance | FO | SE_DDC |
| 99/1000 | Dependence Entropy | GLDM | b_2500_ |
| 98/1000 | Dependence Non-Uniformity Normalized | GLDM | BE_IVIM_D |
| 98/1000 | Dependence Non-Uniformity | GLDM | DKI_K |
| 97/1000 | Gray Level Non-Uniformity | GLDM | b_2500_ |
| 96/1000 | Small Dependence High Gray Level Emphasis | GLDM | b_2500_ |
| 95/1000 | Inverse Variance | GLCM | ME_ADC _all_b_ |
| 93/1000 | Skewness | FO | BE_IVIM_D |
| 92/1000 | Autocorrelation | GLCM | BE_IVIM_D |
| 92/1000 | Gray Level Variance | GLSZM | BE_IVIM_D |
| 92/1000 | Mean | FO | DKI_K |
| 91/1000 | Size Zone Non-Uniformity | GLSZM | DKI_K |
| 88/1000 | Dependence Non-Uniformity Normalized | GLDM | SE_DDC |
| 88/1000 | Low Gray Level Zone Emphasis | GLSZM | DKI_K |
| 87/1000 | 90 Percentile | FO | SE_DDC |
| 87/1000 | Difference Entropy | GLCM | SE_DDC |
| 86/1000 | Imc2 | GLCM | b_2500_ |
| 83/1000 | Sum Squares | GLCM | SE_DDC |
| 82/1000 | High Gray Level Run Emphasis | GLRLM | b_2500_ |
| 81/1000 | Difference Variance | GLCM | ME_ADC _all_b_ |
| 81/1000 | Difference Entropy | GLCM | DKI_K |
| 81/1000 | Run Length Non-Uniformity | GLRLM | DKI_K |
| 79/1000 | Large Dependence Low Gray Level Emphasis | GLDM | SE_DDC |
| 78/1000 | Dependence Non-Uniformity | GLDM | ME_ADC _all_b_ |
| 78/1000 | Small Area Low Gray Level Emphasis | GLSZM | BE_IVIM_D |
| 77/1000 | Run Percentage | GLRLM | SE_DDC |
| 76/1000 | Imc2 | GLCM | BE_IVIM_D |
| 76/1000 | Inverse Variance | GLCM | DKI_K |
| 75/1000 | Sum Squares | GLCM | DKI_K |
| 74/1000 | Median | FO | b_2500_ |
| 74/1000 | Imc1 | GLCM | DKI_K |
| 73/1000 | Correlation | GLCM | ME_ADC _all_b_ |
| 73/1000 | Size Zone Non-Uniformity Normalized | GLSZM | DKI_K |
| 72/1000 | High Gray Level Zone Emphasis | GLSZM | b_2500_ |
| 72/1000 | Cluster Tendency | GLCM | ME_ADC _all_b_ |
| 72/1000 | Median | FO | DKI_K |
| 71/1000 | Inverse Variance | GLCM | BE_IVIM_D |
| 71/1000 | Run Length Non-Uniformity | GLRLM | BE_IVIM_D |
| 70/1000 | Uniformity | FO | DKI_K |
| 68/1000 | Size Zone Non-Uniformity Normalized | GLSZM | BE_IVIM_D |
| 67/1000 | Maximum 3D Diameter | Shape | All |
| 67/1000 | Low Gray Level Zone Emphasis | GLSZM | SE_DDC |
| 65/1000 | Autocorrelation | GLCM | ME_ADC _all_b_ |
| 65/1000 | Joint Average | GLCM | BE_IVIM_D |
| 64/1000 | Total Energy | FO | SE_DDC |
| 63/1000 | Robust Mean Absolute Deviation | FO | ME_ADC _all_b_ |
| 63/1000 | Dependence Entropy | GLDM | ME_ADC _all_b_ |
| 62/1000 | Cluster Tendency | GLCM | BE_IVIM_D |
| 61/1000 | Gray Level Variance | GLRLM | b_2500_ |
| 61/1000 | Dependence Variance | GLDM | ME_ADC _all_b_ |
| 60/1000 | Joint Average | GLCM | b_2500_ |
| 60/1000 | Dependence Variance | GLDM | DKI_K |
| 58/1000 | Large Dependence Emphasis | GLDM | BE_IVIM_D |
| 57/1000 | Id | GLCM | BE_IVIM_D |
| 57/1000 | Mean Absolute Deviation | FO | SE_DDC |
| 57/1000 | Large Area High Gray Level Emphasis | GLSZM | DKI_K |
| 55/1000 | Short Run High Gray Level Emphasis | GLRLM | ME_ADC _all_b_ |
| 54/1000 | Entropy | FO | ME_ADC _all_b_ |
| 54/1000 | Low Gray Level Zone Emphasis | GLSZM | ME_ADC _all_b_ |
| 53/1000 | Sum Squares | GLCM | BE_IVIM_D |
| 52/1000 | Small Area High Gray Level Emphasis | GLSZM | BE_IVIM_D |
| 51/1000 | Cluster Shade | GLCM | SE_DDC |
| 50/1000 | Joint Entropy | GLCM | BE_IVIM_D |
| 50/1000 | Gray Level Non-Uniformity Normalized | GLSZM | DKI_K |
| 49/1000 | Run Entropy | GLRLM | BE_IVIM_D |
| 48/1000 | Large Dependence Low Gray Level Emphasis | GLDM | ME_ADC _all_b_ |
| 46/1000 | Gray Level Non-Uniformity | GLRLM | DKI_K |
| 44/1000 | Id | GLCM | DKI_K |
| 43/1000 | High Gray Level Run Emphasis | GLRLM | DKI_K |
| 42/1000 | Small Dependence Low Gray Level Emphasis | GLDM | ME_ADC _all_b_ |
| 42/1000 | Small Dependence High Gray Level Emphasis | GLDM | BE_IVIM_D |
| 41/1000 | Energy | FO | b_2500_ |
| 40/1000 | Run Length Non-Uniformity | GLRLM | b_2500_ |
| 40/1000 | Run Length Non-Uniformity Normalized | GLRLM | ME_ADC _all_b_ |
| 40/1000 | Gray Level Variance | GLRLM | DKI_K |
| 39/1000 | Size Zone Non-Uniformity Normalized | GLSZM | ME_ADC _all_b_ |
| 38/1000 | Gray Level Non-Uniformity | GLSZM | ME_ADC _all_b_ |
| 37/1000 | Cluster Shade | GLCM | ME_ADC _all_b_ |
| 37/1000 | Joint Entropy | GLCM | DKI_K |
| 35/1000 | Gray Level Variance | GLSZM | ME_ADC _all_b_ |
| 35/1000 | Range | FO | SE_DDC |
| 34/1000 | Root Mean Squared | FO | SE_DDC |
| 33/1000 | Low Gray Level Emphasis | GLDM | ME_ADC _all_b_ |
| 33/1000 | High Gray Level Run Emphasis | GLRLM | BE_IVIM_D |
| 33/1000 | Gray Level Non-Uniformity | GLRLM | SE_DDC |
| 33/1000 | Large Area Low Gray Level Emphasis | GLSZM | DKI_K |
| 30/1000 | Long Run High Gray Level Emphasis | GLRLM | ME_ADC _all_b_ |
| 30/1000 | Cluster Tendency | GLCM | SE_DDC |
| 30/1000 | Small Area High Gray Level Emphasis | GLSZM | DKI_K |
| 30/1000 | Zone Percentage | GLSZM | DKI_K |
| 29/1000 | Large Area High Gray Level Emphasis | GLSZM | ME_ADC _all_b_ |
| 29/1000 | Low Gray Level Run Emphasis | GLRLM | SE_DDC |
| 28/1000 | Cluster Prominence | GLCM | b_2500_ |
| 28/1000 | Low Gray Level Run Emphasis | GLRLM | ME_ADC _all_b_ |
| 28/1000 | Small Dependence High Gray Level Emphasis | GLDM | ME_ADC _all_b_ |
| 28/1000 | Long Run High Gray Level Emphasis | GLRLM | DKI_K |
| 27/1000 | Gray Level Non-Uniformity Normalized | GLRLM | b_2500_ |
| 27/1000 | Run Length Non-Uniformity Normalized | GLRLM | DKI_K |
| 27/1000 | Zone Variance | GLSZM | DKI_K |
| 25/1000 | Long Run Emphasis | GLRLM | ME_ADC _all_b_ |
| 25/1000 | Gray Level Non-Uniformity | GLDM | ME_ADC _all_b_ |
| 25/1000 | Minimum | FO | DKI_K |
| 24/1000 | Gray Level Non-Uniformity | GLSZM | BE_IVIM_D |
| 23/1000 | Mesh Volume | Shape | All |
| 23/1000 | Autocorrelation | GLCM | SE_DDC |
| 23/1000 | 90 Percentile | FO | DKI_K |
| 22/1000 | Short Run Emphasis | GLRLM | SE_DDC |
| 21/1000 | Id | GLCM | b_2500_ |
| 21/1000 | Zone Variance | GLSZM | b_2500_ |
| 21/1000 | Gray Level Non-Uniformity | GLRLM | BE_IVIM_D |
| 20/1000 | Gray Level Variance | GLRLM | ME_ADC _all_b_ |
| 19/1000 | Long Run Emphasis | GLRLM | BE_IVIM_D |
| 19/1000 | Zone Variance | GLSZM | BE_IVIM_D |
| 19/1000 | Small Area Low Gray Level Emphasis | GLSZM | SE_DDC |
| 18/1000 | Gray Level Non-Uniformity Normalized | GLRLM | ME_ADC _all_b_ |
| 18/1000 | Difference Average | GLCM | SE_DDC |
| 17/1000 | High Gray Level Emphasis | GLDM | b_2500_ |
| 16/1000 | Small Area Emphasis | GLSZM | ME_ADC _all_b_ |
| 15/1000 | Robust Mean Absolute Deviation | FO | SE_DDC |
| 15/1000 | Gray Level Non-Uniformity Normalized | GLSZM | SE_DDC |
| 14/1000 | Dependence Non-Uniformity | GLDM | b_2500_ |
| 14/1000 | Minimum | FO | SE_DDC |
| 13/1000 | Gray Level Variance | GLDM | b_2500_ |
| 13/1000 | Zone Variance | GLSZM | ME_ADC _all_b_ |
| 13/1000 | Gray Level Non-Uniformity | GLSZM | SE_DDC |
| 13/1000 | Run Variance | GLRLM | DKI_K |
| 12/1000 | Run Variance | GLRLM | ME_ADC _all_b_ |
| 11/1000 | Run Percentage | GLRLM | BE_IVIM_D |
| 11/1000 | Idmn | GLCM | SE_DDC |
| 11/1000 | Small Area Emphasis | GLSZM | SE_DDC |
| 10/1000 | Voxel Volume | Shape | All |
| 10/1000 | Run Length Non-Uniformity | GLRLM | ME_ADC _all_b_ |
| 10/1000 | Imc2 | GLCM | SE_DDC |
| 10/1000 | Run Entropy | GLRLM | SE_DDC |
| 10/1000 | Zone Entropy | GLSZM | DKI_K |
| 9/1000 | Sum Entropy | GLCM | b_2500_ |
| 9/1000 | High Gray Level Emphasis | GLDM | ME_ADC _all_b_ |
| 9/1000 | Run Percentage | GLRLM | DKI_K |
| 8/1000 | Gray Level Non-Uniformity Normalized | GLRLM | SE_DDC |
| 8/1000 | Zone Percentage | GLSZM | SE_DDC |
| 8/1000 | Small Area Low Gray Level Emphasis | GLSZM | DKI_K |
| 7/1000 | High Gray Level Run Emphasis | GLRLM | ME_ADC _all_b_ |
| 7/1000 | Short Run Emphasis | GLRLM | ME_ADC _all_b_ |
| 7/1000 | High Gray Level Run Emphasis | GLRLM | SE_DDC |
| 7/1000 | Large Area High Gray Level Emphasis | GLSZM | SE_DDC |
| 7/1000 | Size Zone Non-Uniformity Normalized | GLSZM | SE_DDC |
| 6/1000 | Dependence Non-Uniformity Normalized | GLDM | ME_ADC _all_b_ |
| 6/1000 | Large Dependence High Gray Level Emphasis | GLDM | BE_IVIM_D |
| 6/1000 | Idn | GLCM | SE_DDC |
| 6/1000 | Maximum Probability | GLCM | SE_DDC |
| 6/1000 | Long Run Low Gray Level Emphasis | GLRLM | SE_DDC |
| 5/1000 | Gray Level Non-Uniformity Normalized | GLSZM | ME_ADC _all_b_ |
| 5/1000 | Large Dependence High Gray Level Emphasis | GLDM | ME_ADC _all_b_ |
| 5/1000 | Inverse Variance | GLCM | SE_DDC |
| 5/1000 | Dependence Non-Uniformity | GLDM | SE_DDC |
| 4/1000 | Surface Area | Shape | All |
| 4/1000 | Run Percentage | GLRLM | ME_ADC _all_b_ |
| 4/1000 | High Gray Level Zone Emphasis | GLSZM | SE_DDC |
| 4/1000 | Large Area Emphasis | GLSZM | SE_DDC |
| 4/1000 | Small Area High Gray Level Emphasis | GLSZM | SE_DDC |
| 4/1000 | Dependence Entropy | GLDM | SE_DDC |
| 4/1000 | Maximum | FO | DKI_K |
| 3/1000 | Joint Average | GLCM | SE_DDC |
| 3/1000 | Run Variance | GLRLM | SE_DDC |
| 3/1000 | Size Zone Non-Uniformity | GLSZM | SE_DDC |
| 2/1000 | Uniformity | FO | SE_DDC |
| 2/1000 | Run Length Non-Uniformity | GLRLM | SE_DDC |
| 2/1000 | Zone Variance | GLSZM | SE_DDC |
| 1/1000 | Total Energy | FO | b_2500_ |
| 1/1000 | Variance | FO | ME_ADC _all_b_ |
| 1/1000 | Idm | GLCM | SE_DDC |
| 1/1000 | Id | GLCM | SE_DDC |
| 1/1000 | Gray Level Variance | GLRLM | SE_DDC |
| 1/1000 | Long Run Emphasis | GLRLM | SE_DDC |
| 1/1000 | Large Area Low Gray Level Emphasis | GLSZM | SE_DDC |

**Appendix S5**

**A Case of 23 image datasets**

**
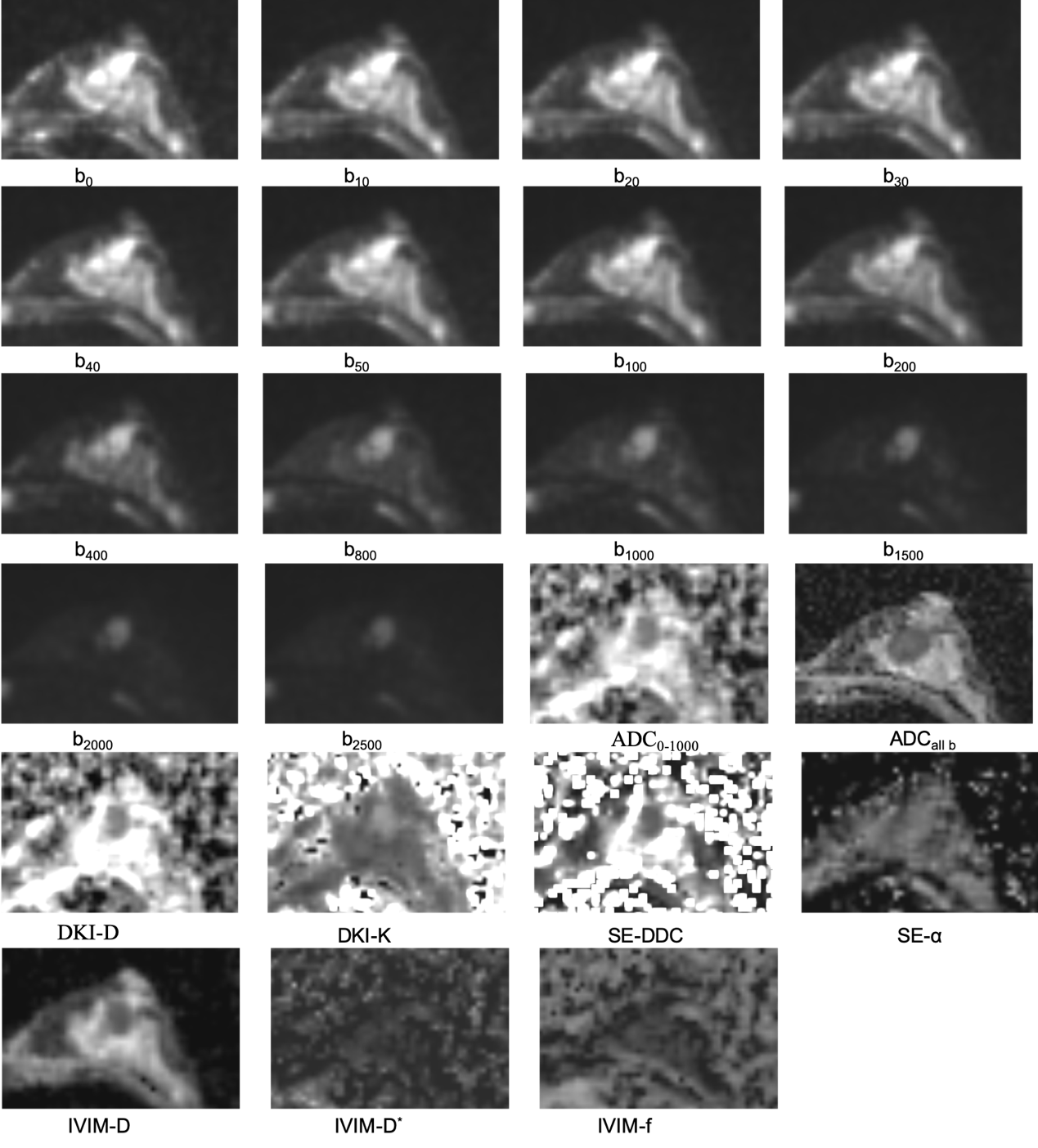
**

Example images of a 53-year-old female with invasive ductal carcinoma in the left breast behind the nipple. The b_0_-b_2500_ images shown a high intensity tumor compared with normal tissues. The ADC_0-1000_, ADC _all b,_ DKI-D, SE-DDC, and IVIM-D images shown a low intensity tumor compared with normal tissues. While on the images of DKI-K, SE-α, IVIM-D*and IVIM-f shown a high intensity tumour compared with normal tissues.

**Appendix S6**

**Comparisons between RF and L1R, PCA, and SVM in the top 5 image sets**

| Maps | RF vs. L1R | RF vs. PCA | RF vs. SVM |
| --- | --- | --- | --- |
|  | *p value* | | |
| ADC_0-1000_ | 0.32 | 0.0019* | 0.77 |
| BE_IVIM_D | 0.23 | 0.0019* | 0.0019* |

*represent significant difference.

**Appendix S7 Top 20 radiomic features of all DWI sequences ranked by the mean decrease in impurity of RF.**


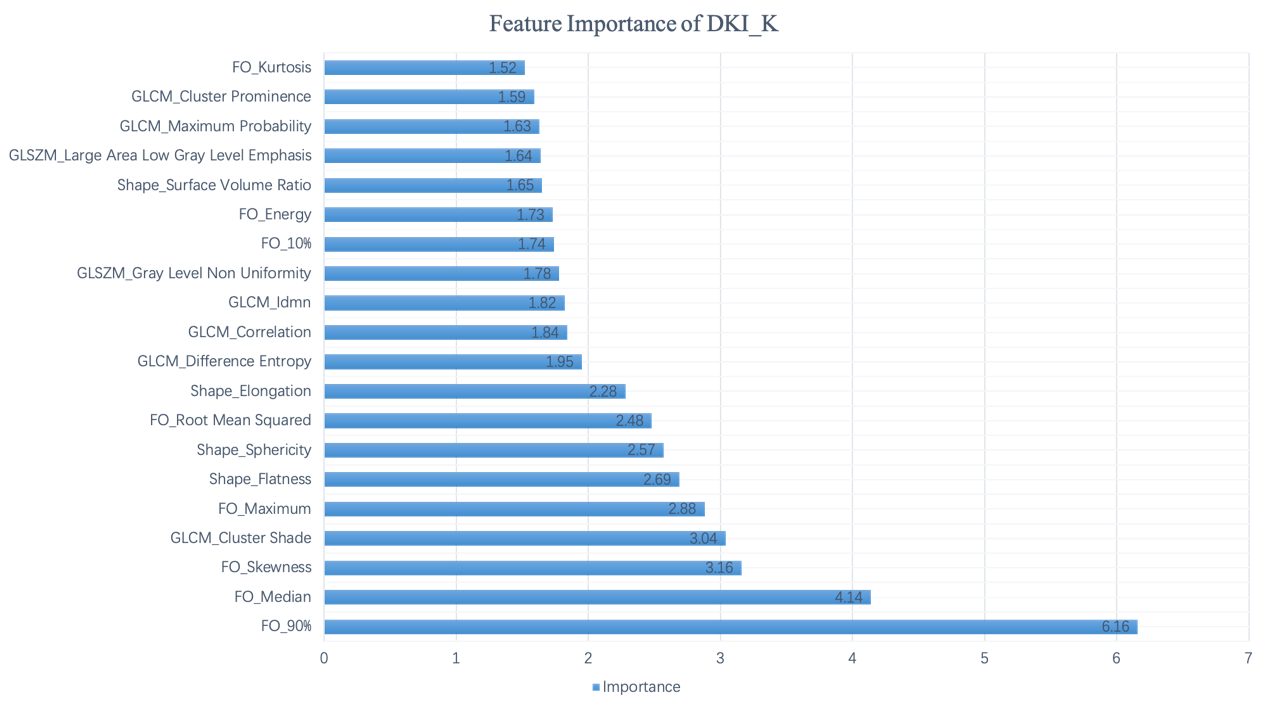


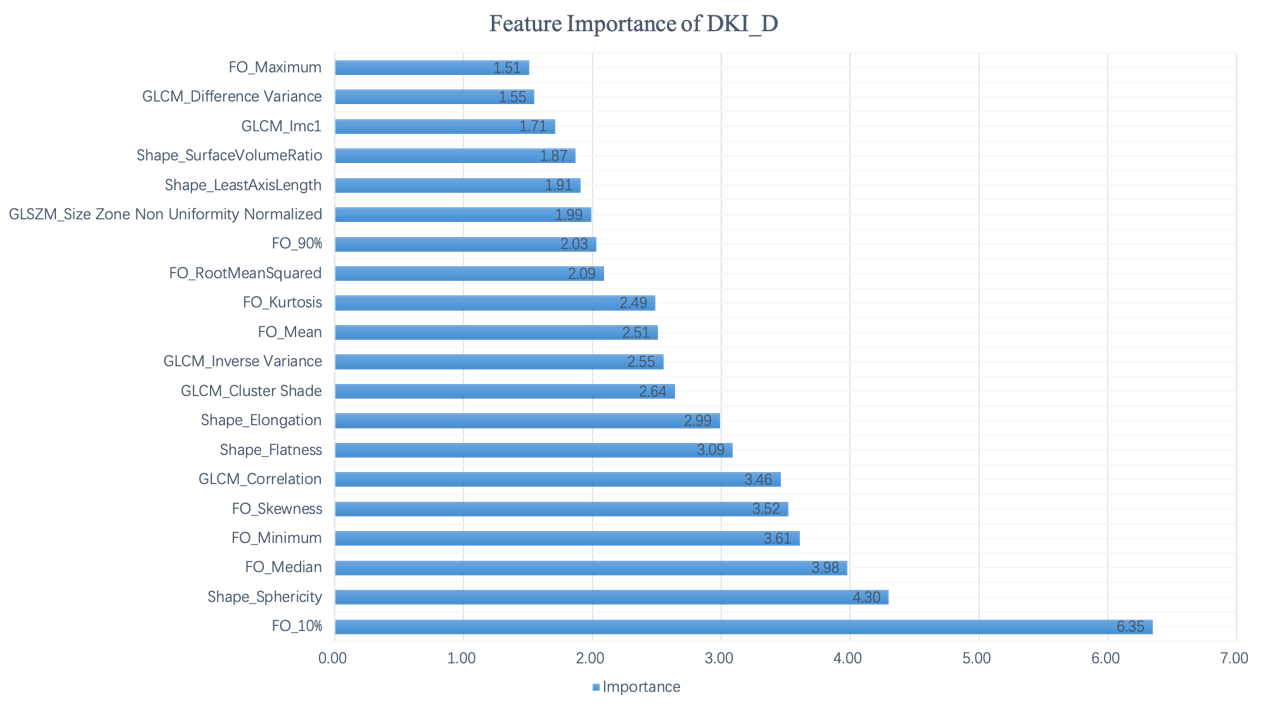


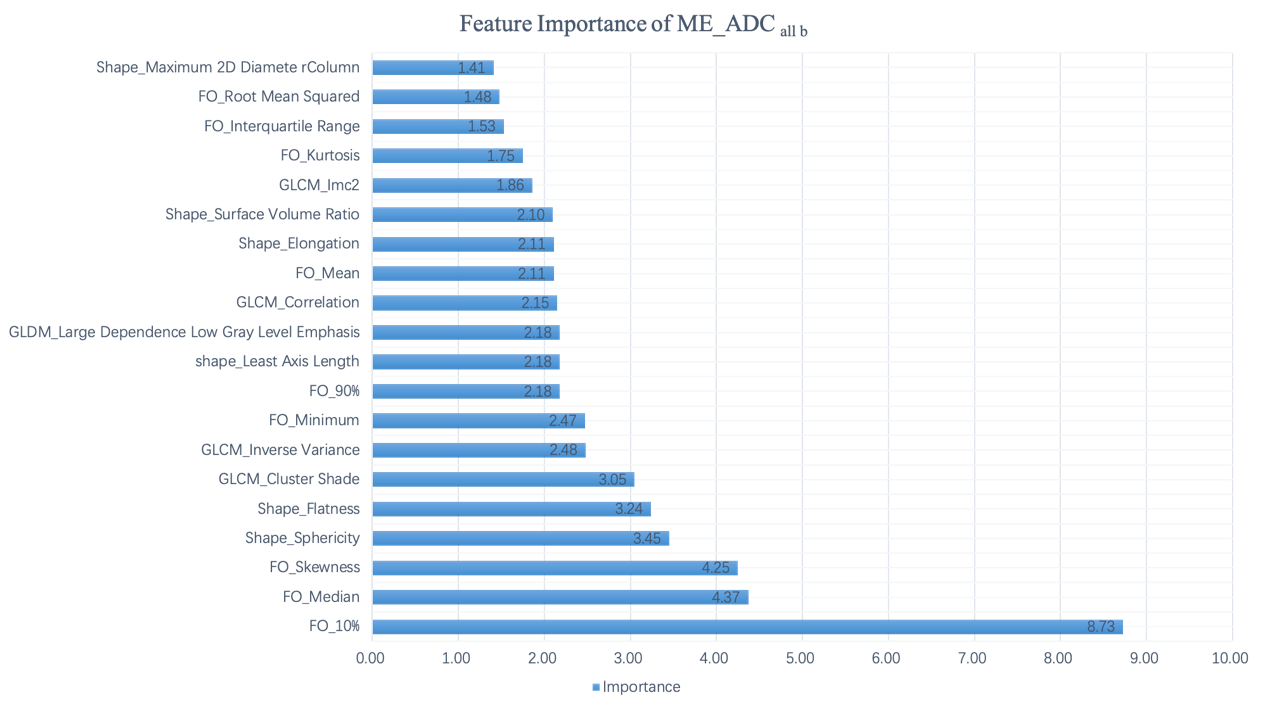


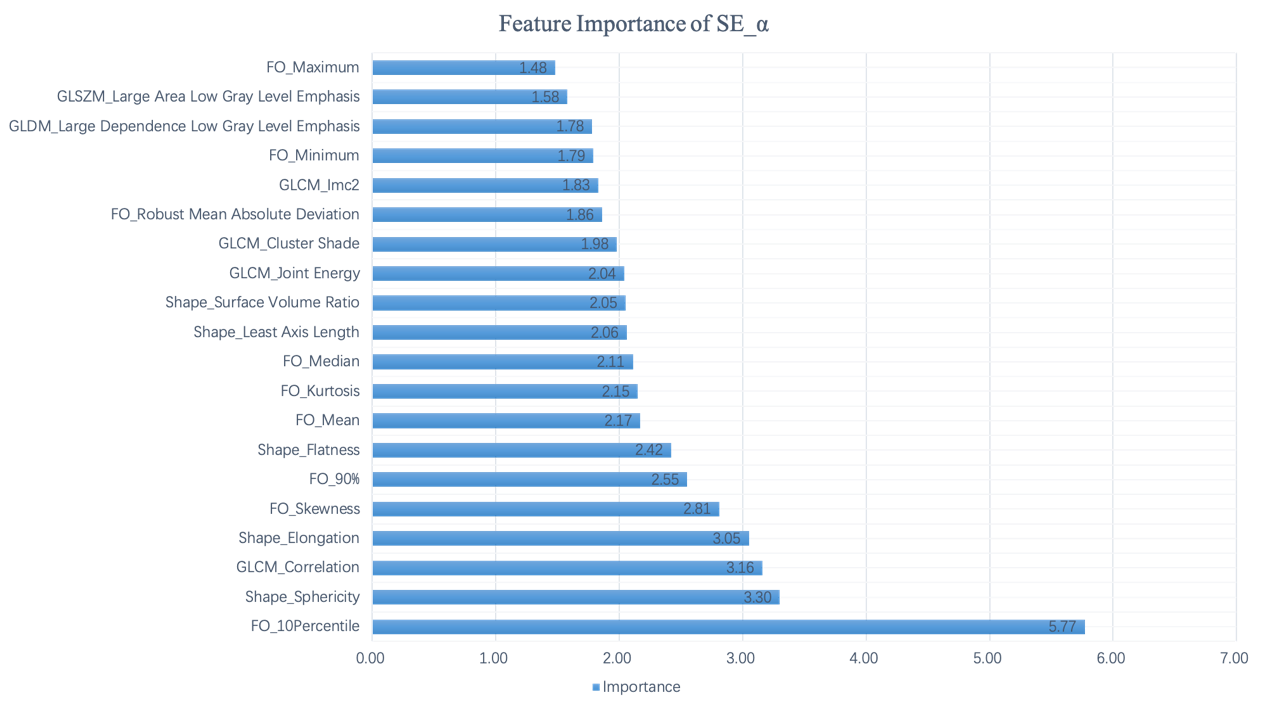


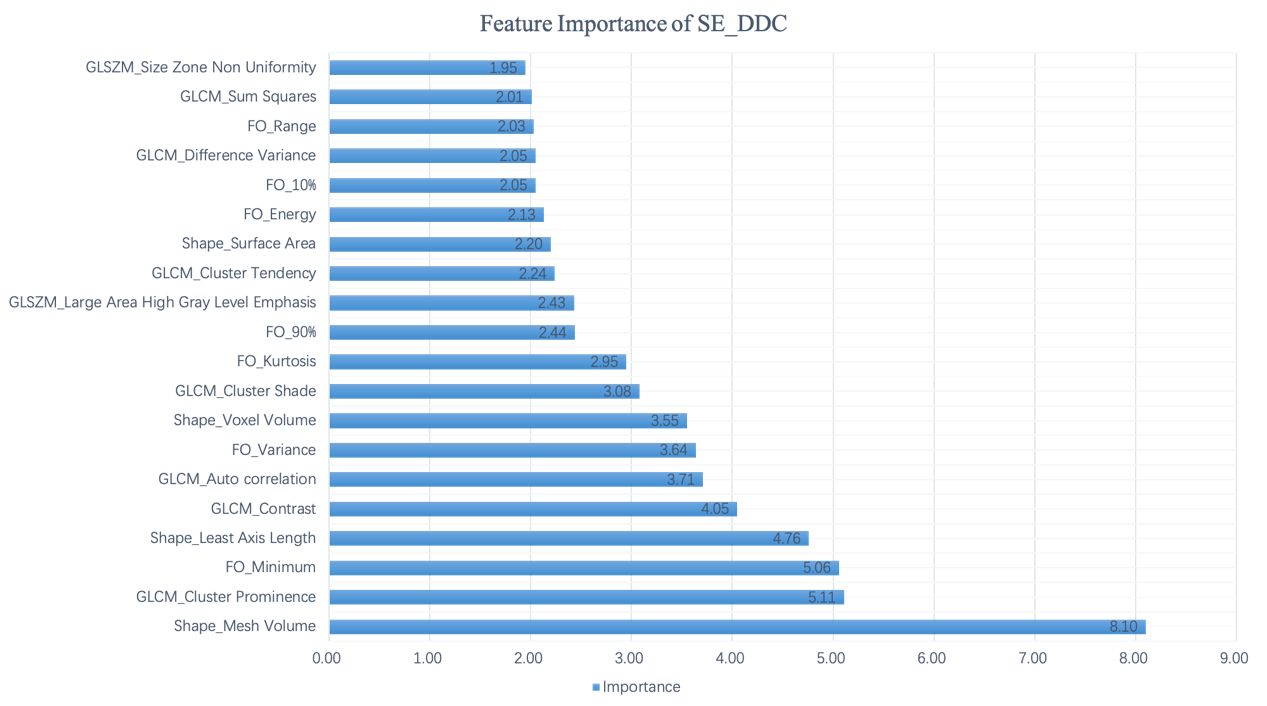


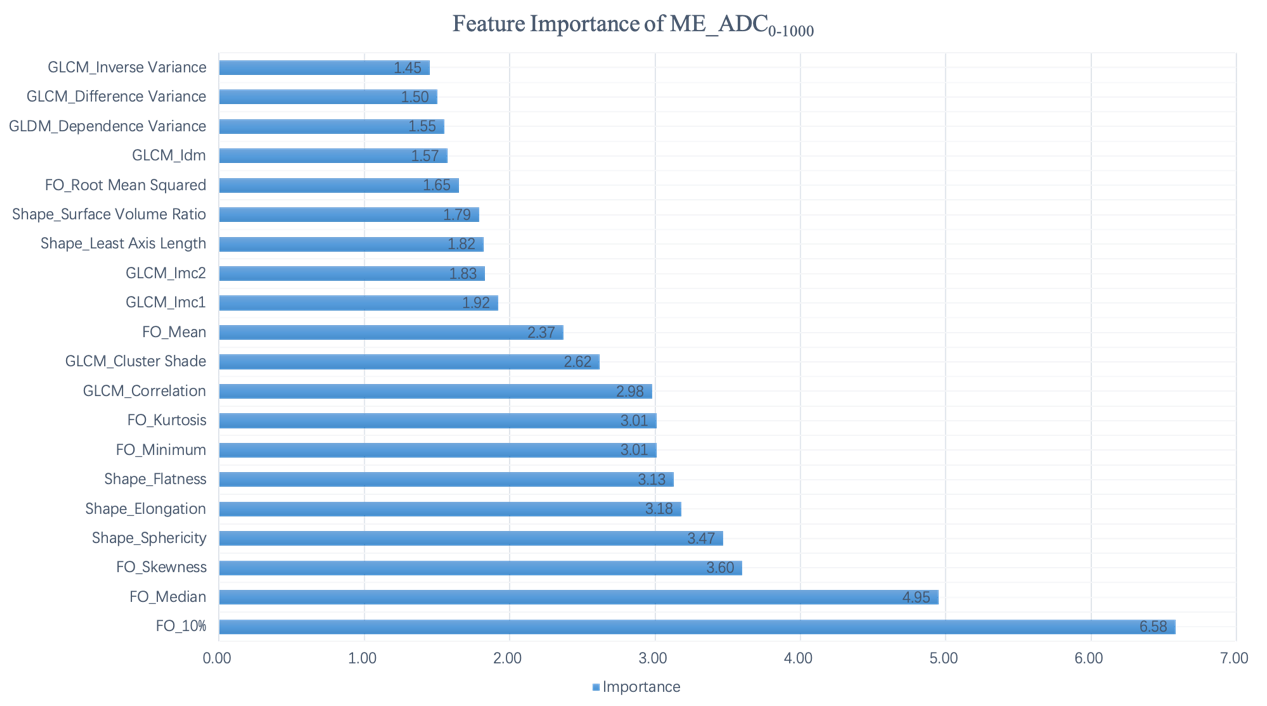


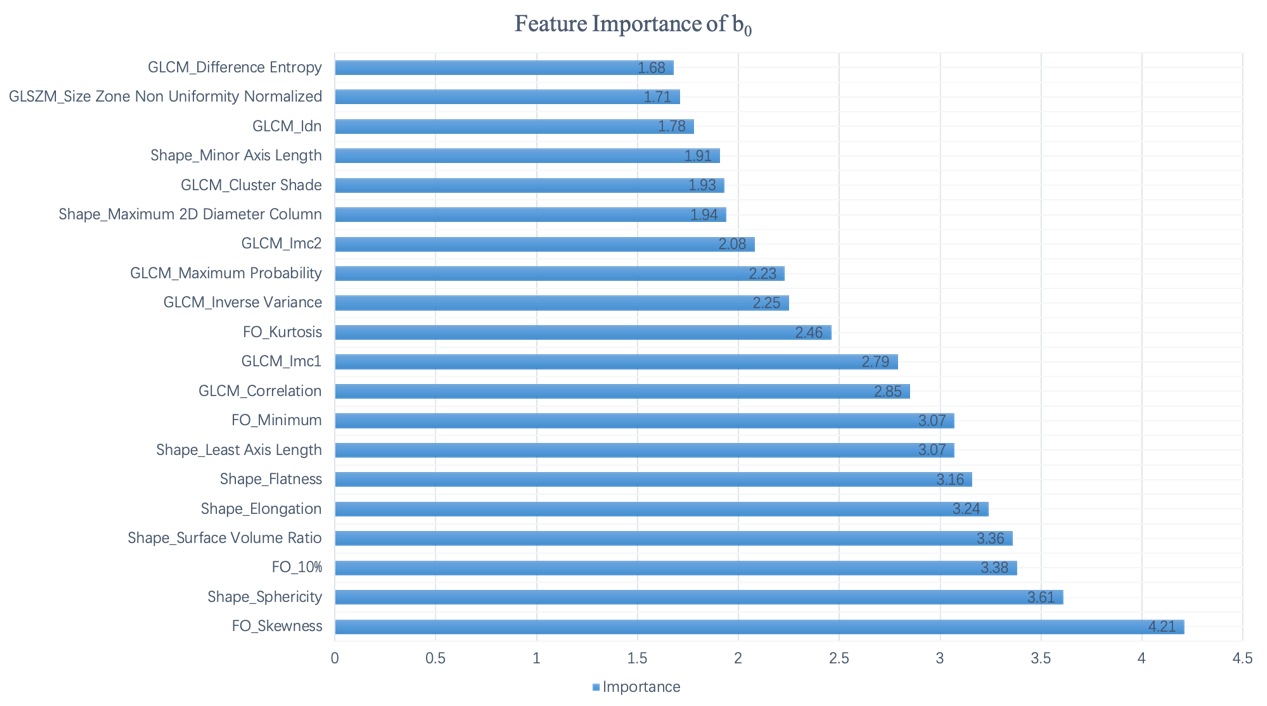


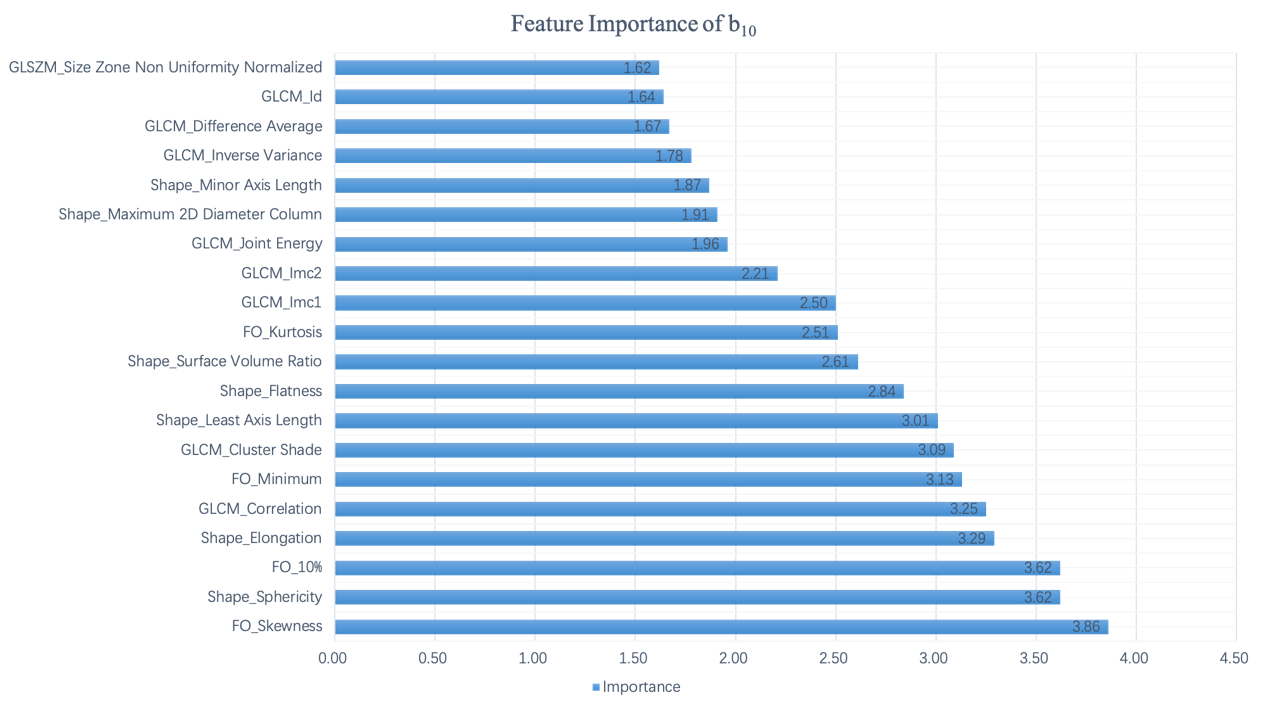


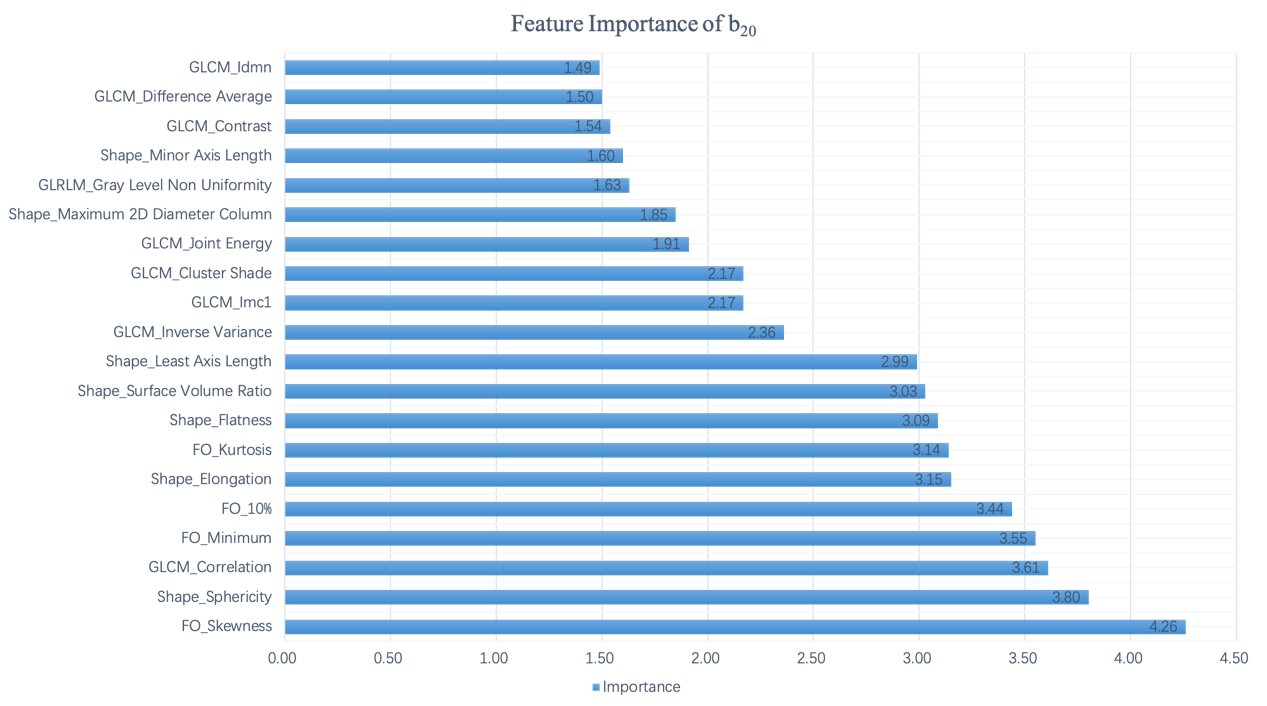


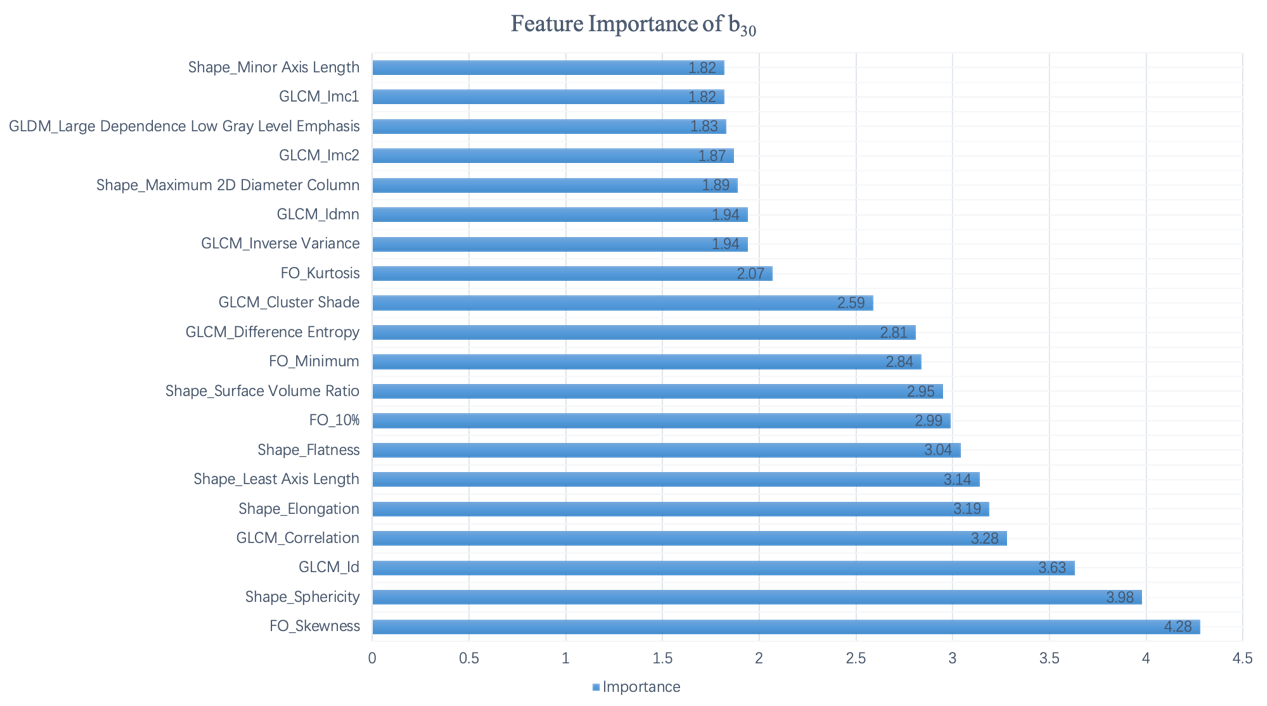


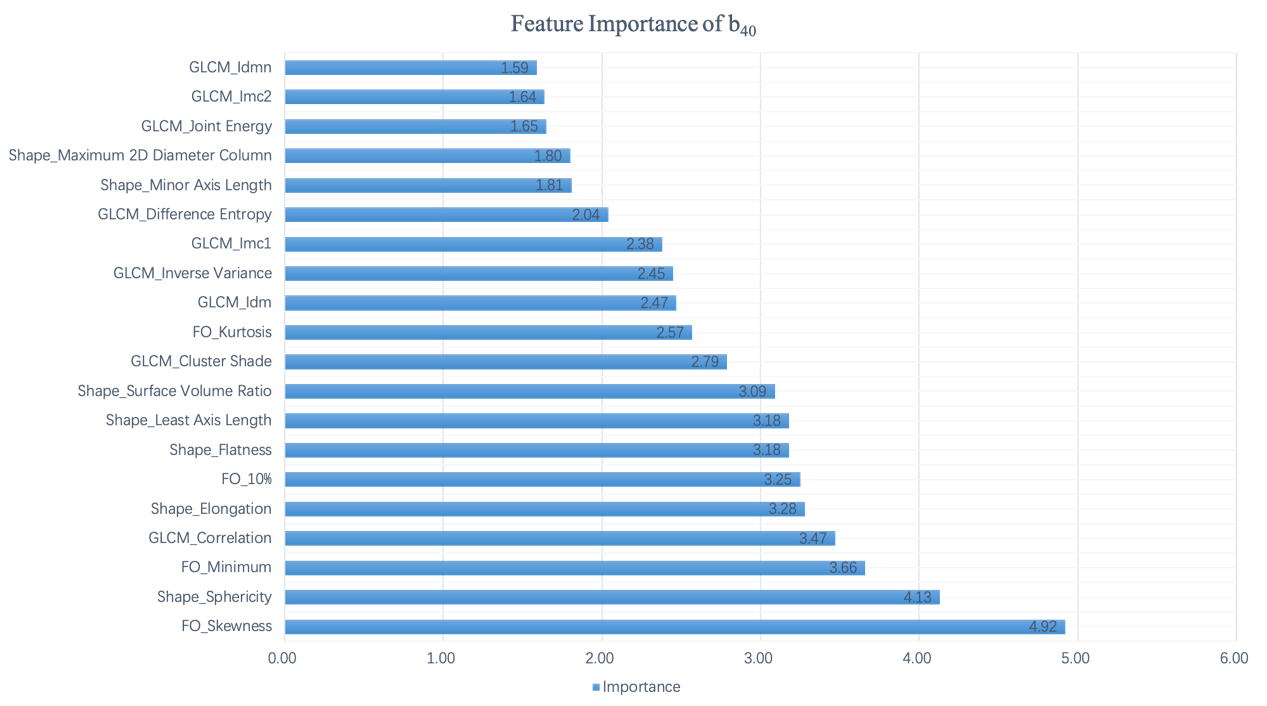


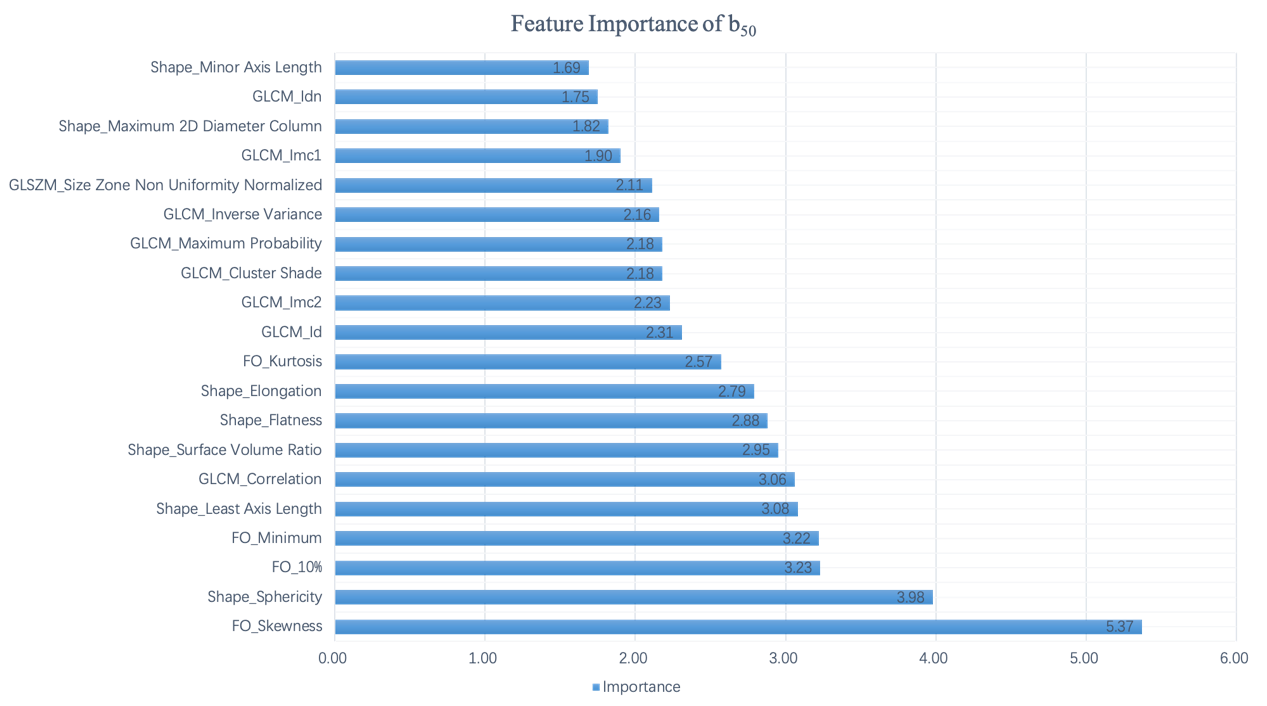


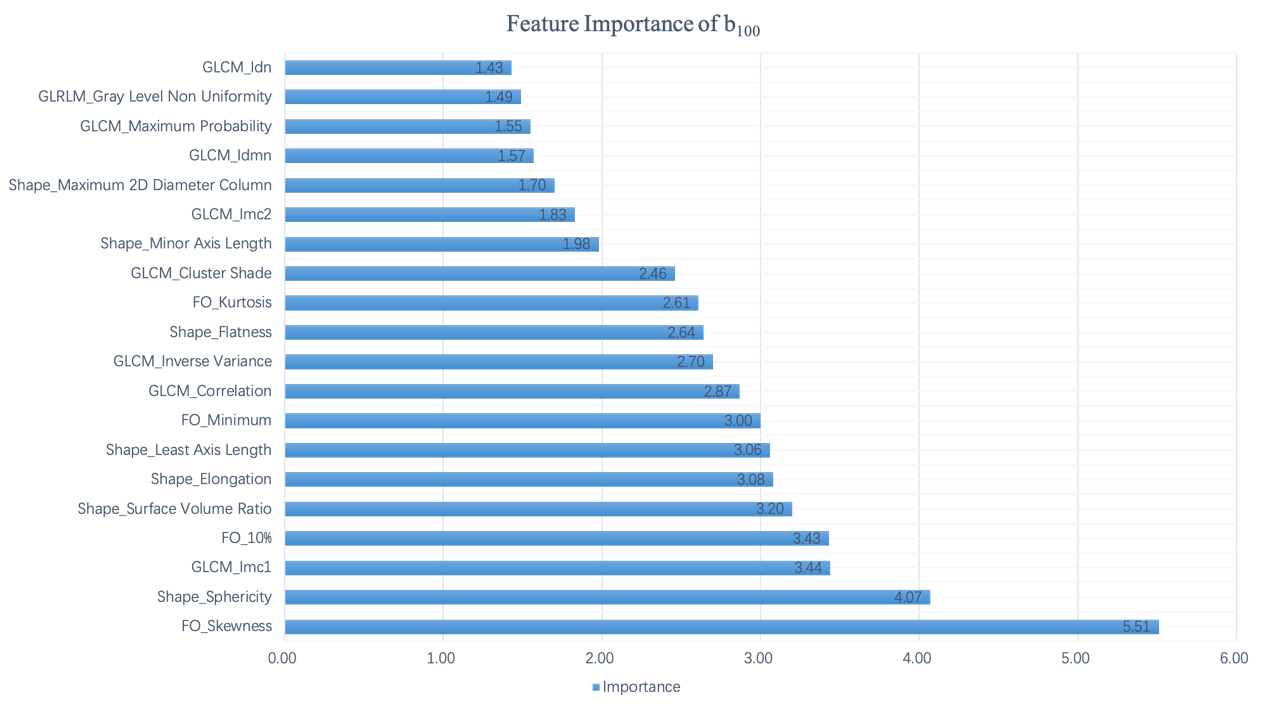


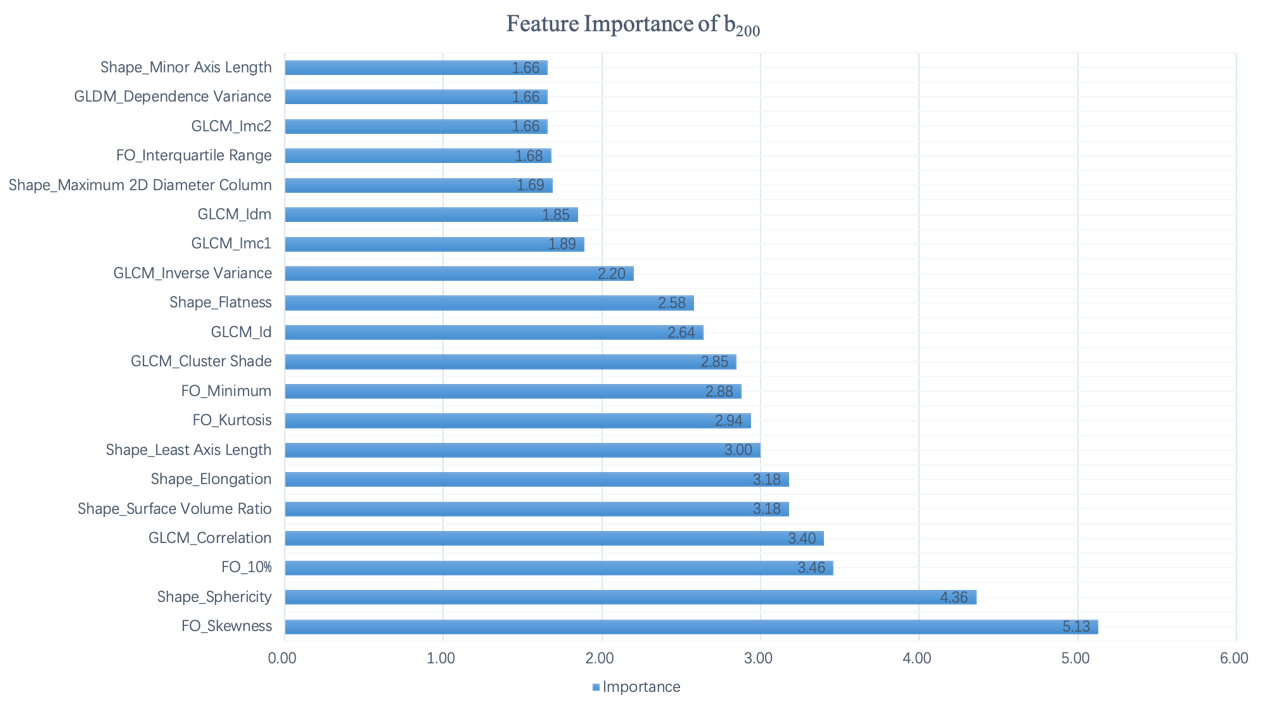


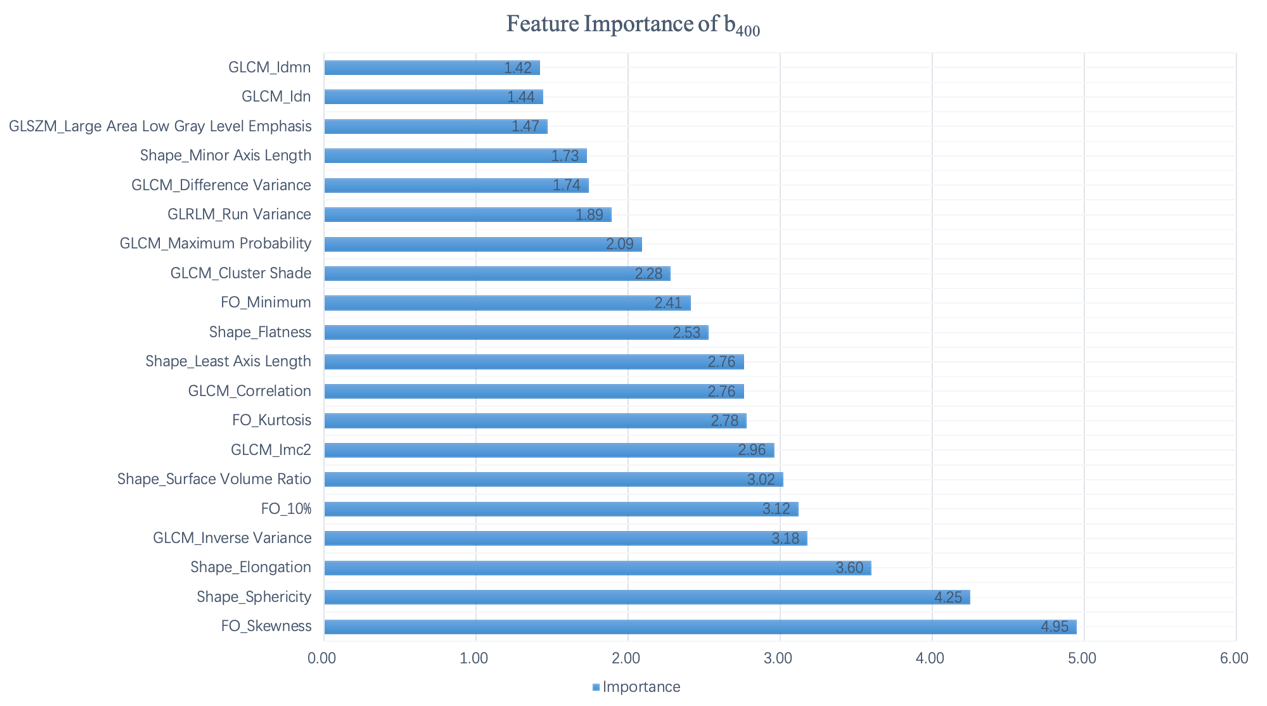


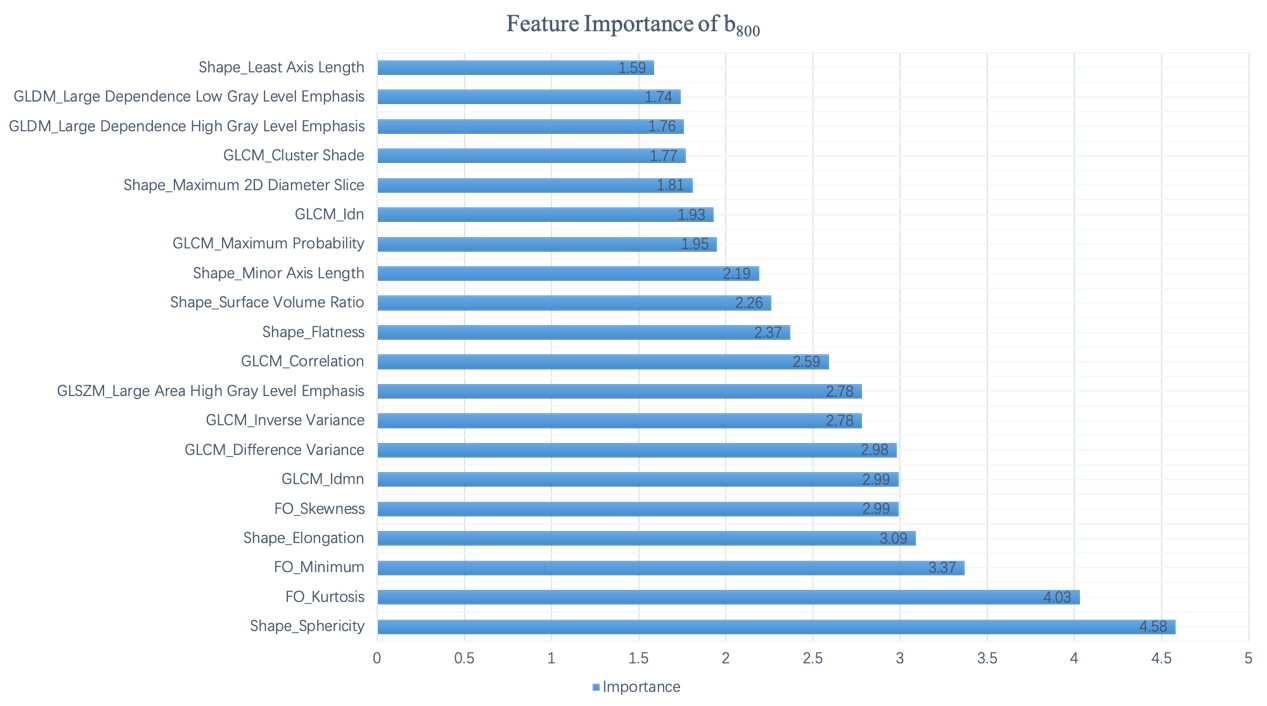


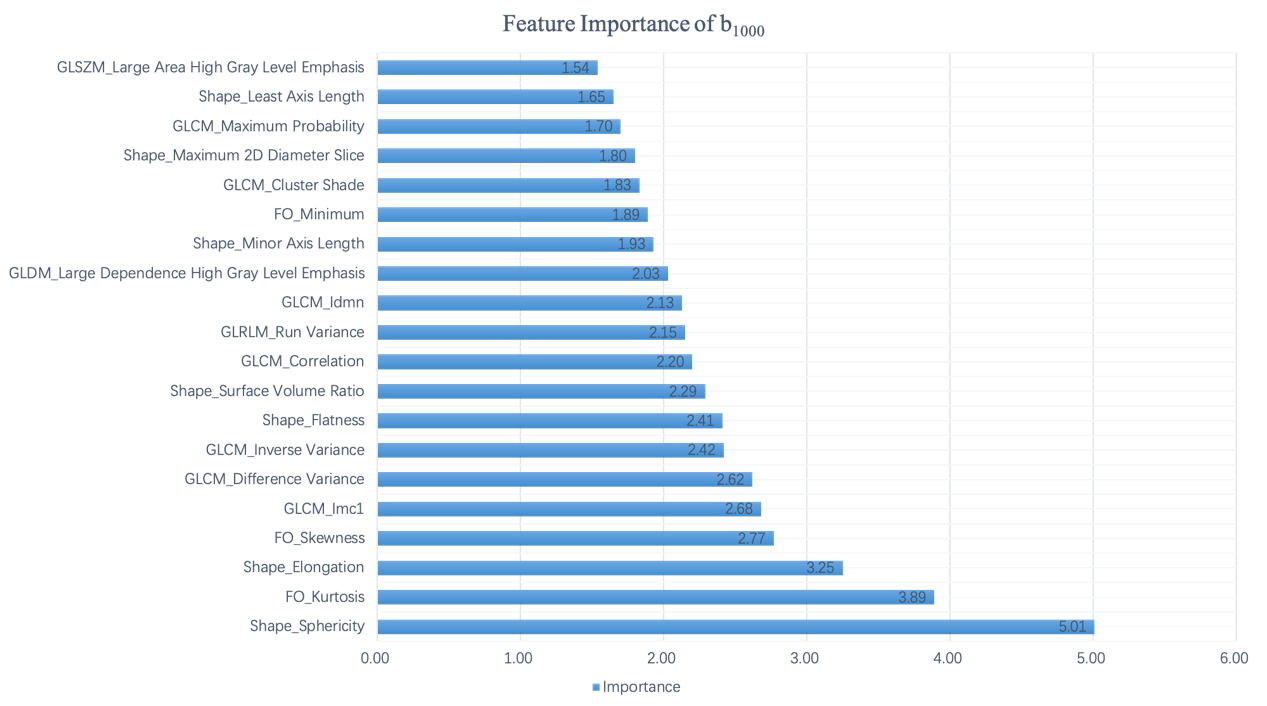


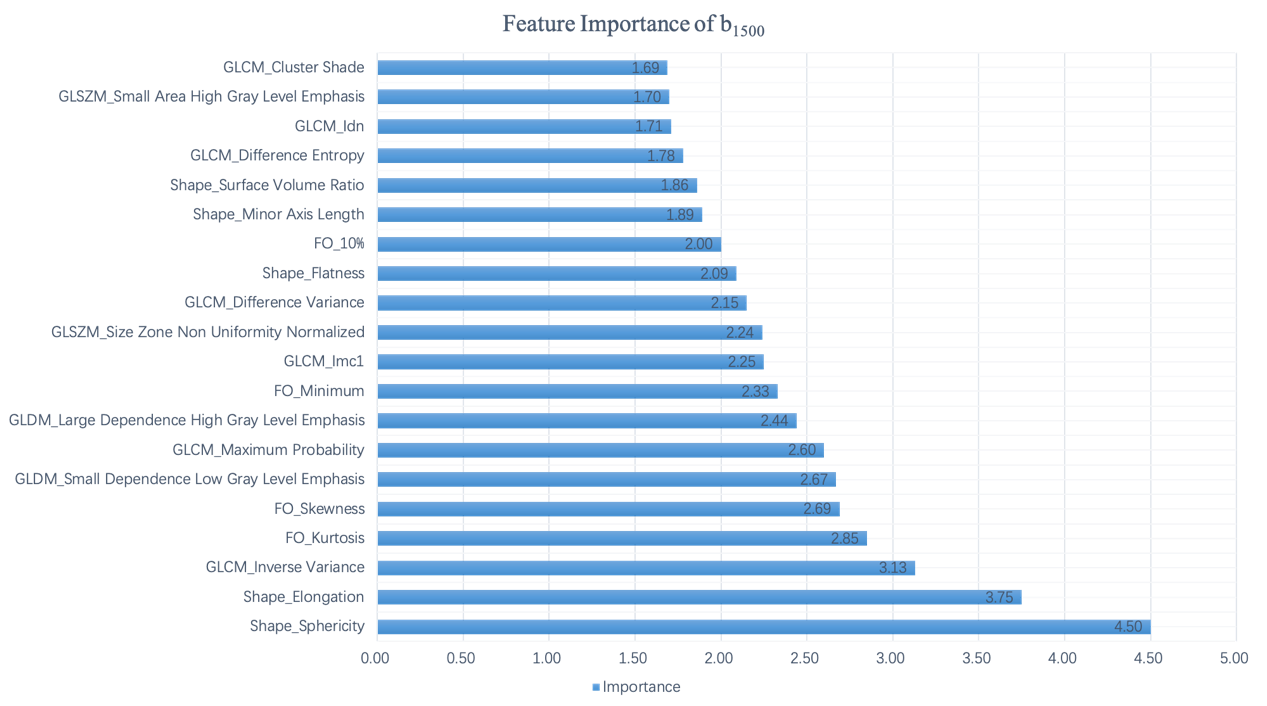


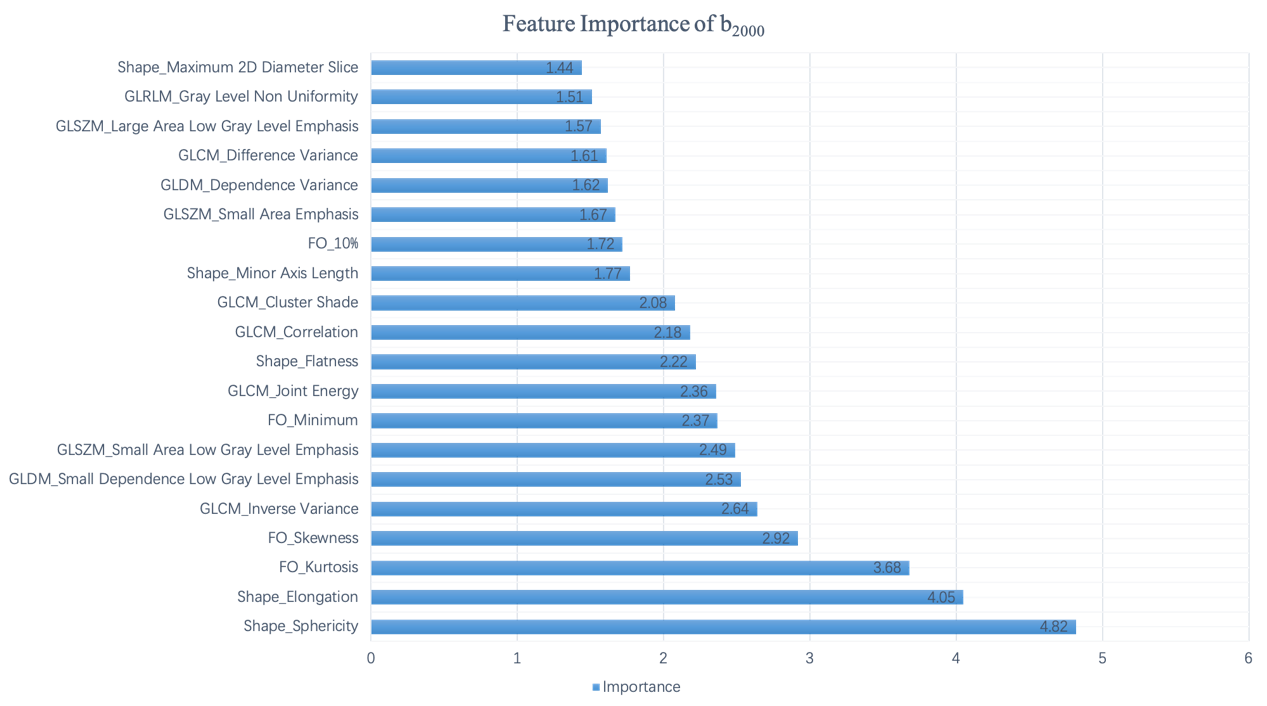


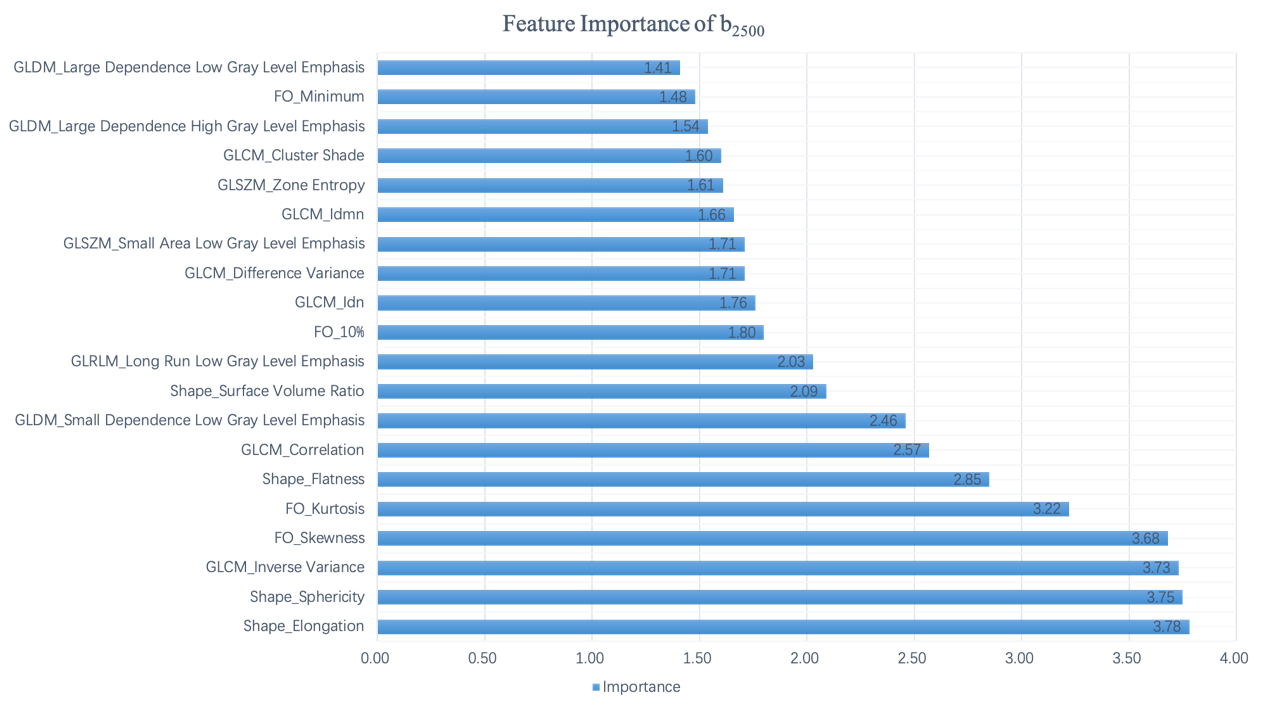


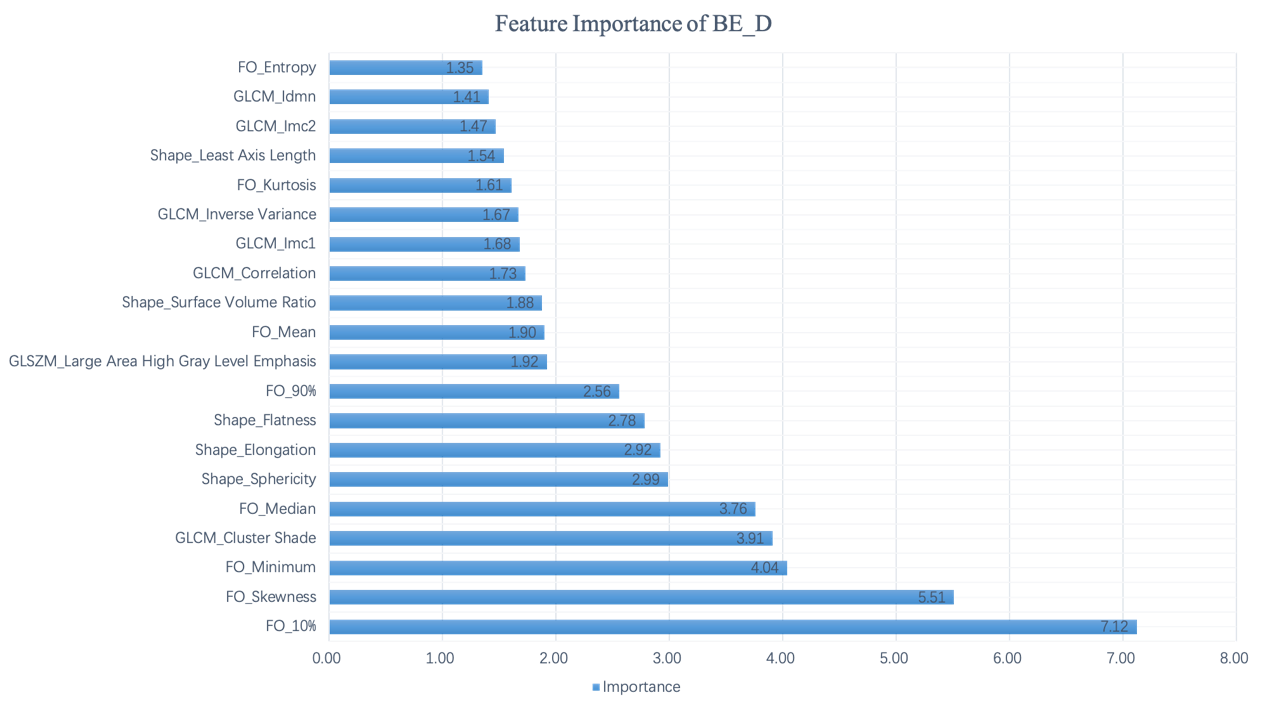


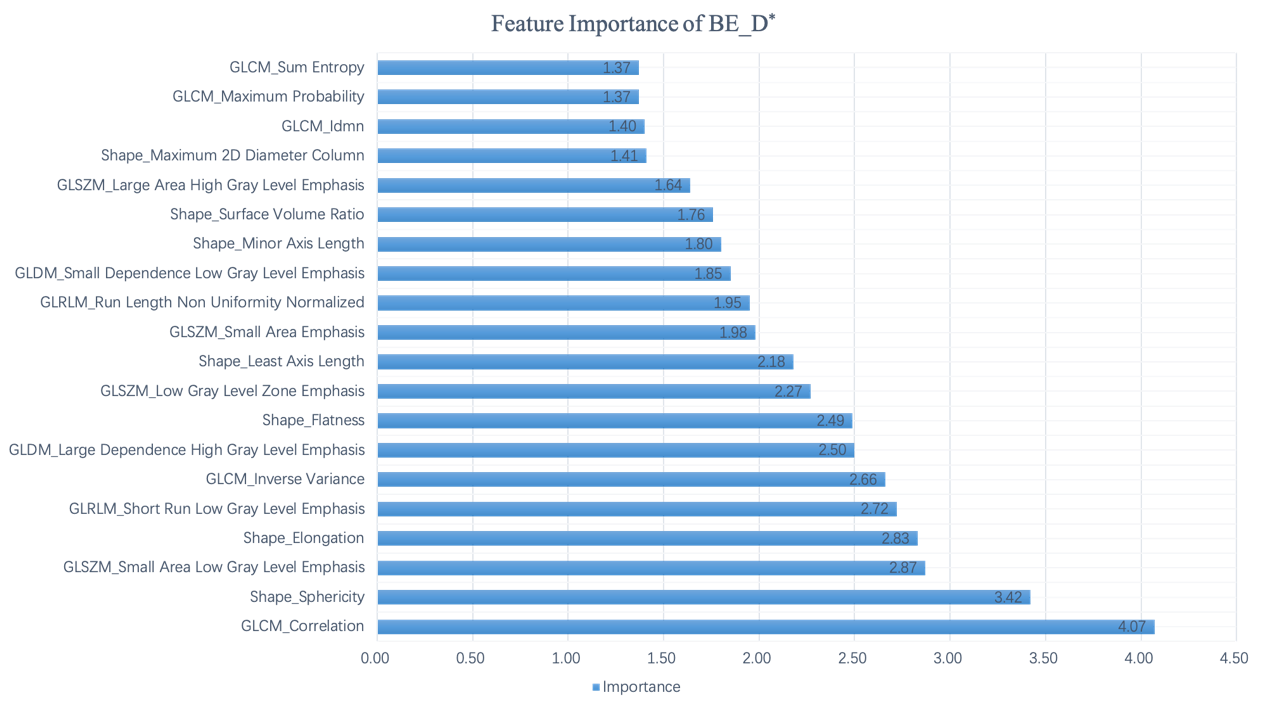


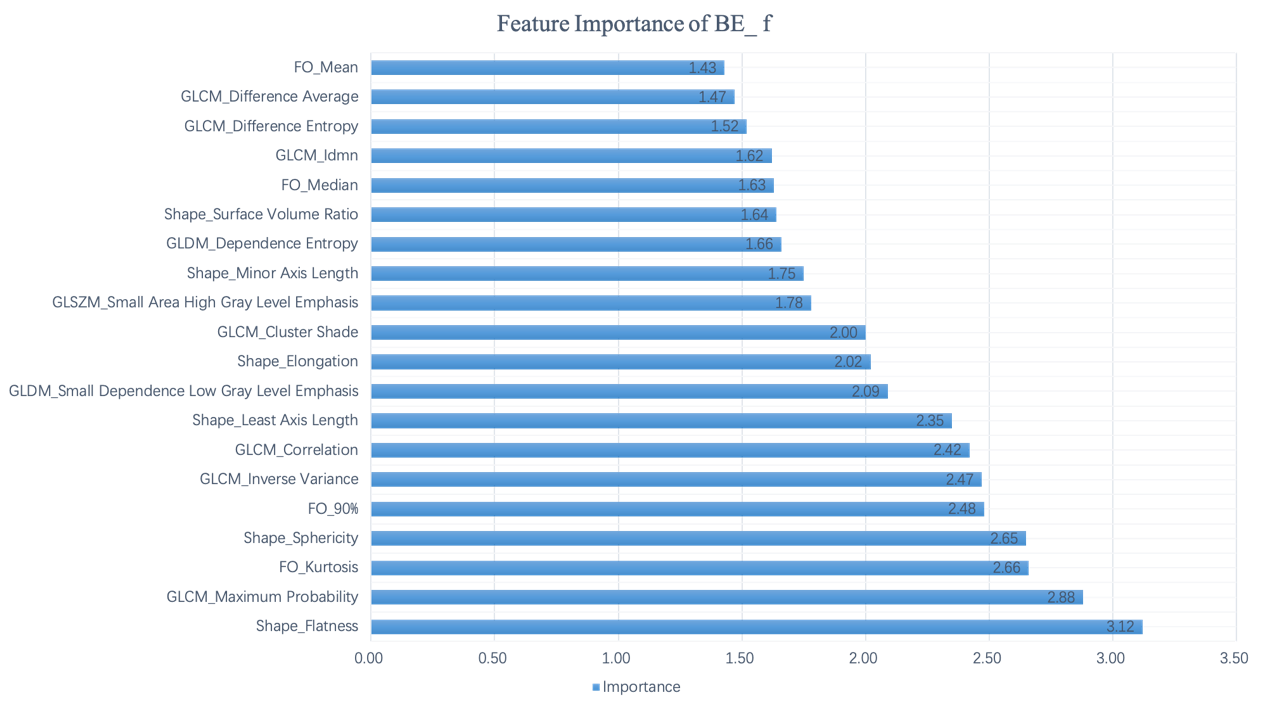


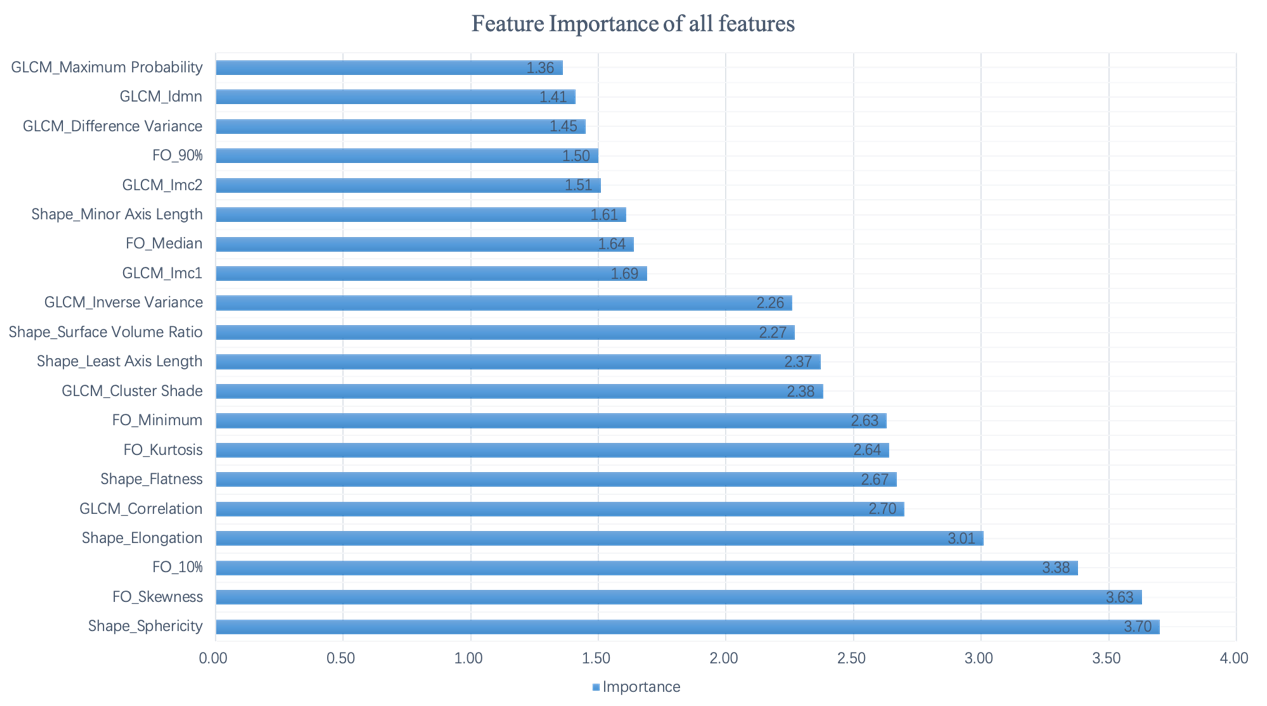


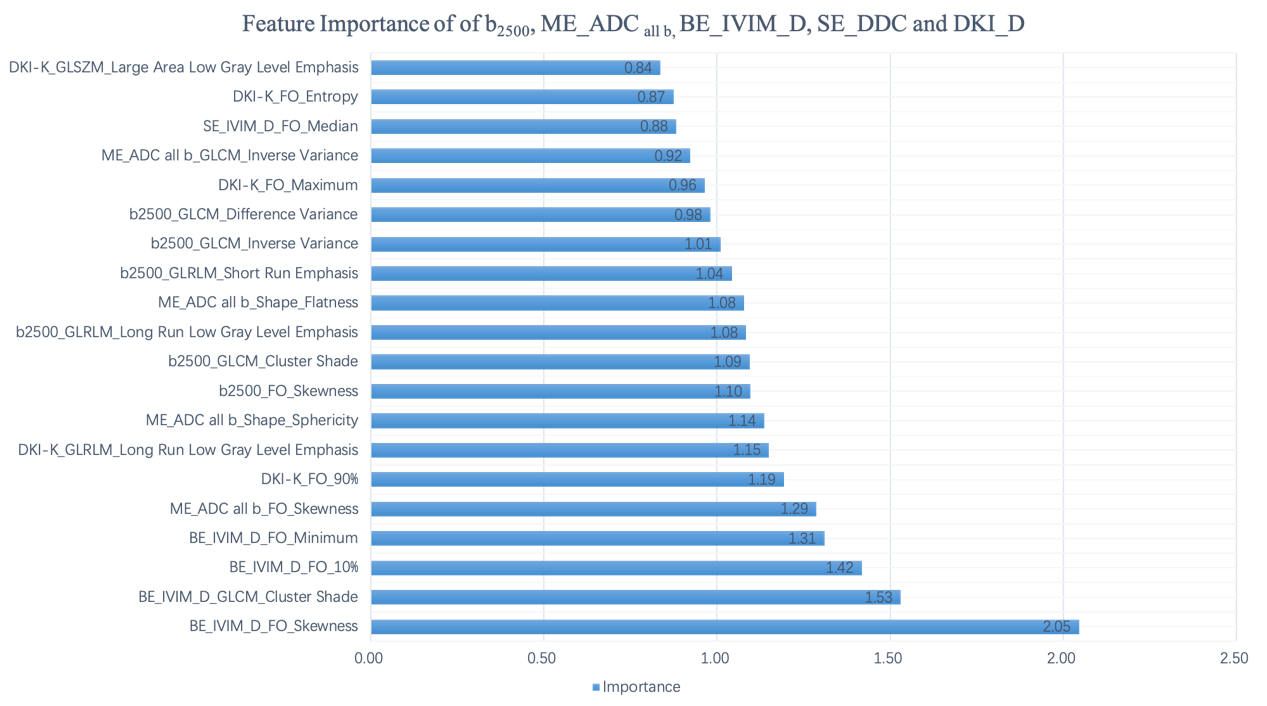

Supplement: Supplementary file 1 — Additional file 1. The scanning parameters of T2WI, multi-b DWI, pre-contrast T1WI, and DCE T1WI. [file 12967_2021_3117_MOESM1_ESM.docx]
